# Extreme multifunctional proteins identified from a human protein interaction network

**DOI:** 10.1038/ncomms8412

**Published:** 2015-06-09

**Authors:** Charles E. Chapple, Benoit Robisson, Lionel Spinelli, Céline Guien, Emmanuelle Becker, Christine Brun

**Affiliations:** 1 Aix-Marseille University, TAGC, Marseille F-13009, France; 2 INSERM UMR_S1090, Marseille F-13009, France; 3 Aix-Marseille University, CIML, Marseille F-13009, France; 4 CNRS, UMR 7280, Marseille F-13009, France; 5 INSERM, U631, Marseille F-13009, France; 6 CNRS, Marseille F-13009, France

## Abstract

Moonlighting proteins are a subclass of multifunctional proteins whose functions are unrelated. Although they may play important roles in cells, there has been no large-scale method to identify them, nor any effort to characterize them as a group. Here, we propose the first method for the identification of ‘extreme multifunctional' proteins from an interactome as a first step to characterize moonlighting proteins. By combining network topological information with protein annotations, we identify 430 extreme multifunctional proteins (3% of the human interactome). We show that the candidates form a distinct sub-group of proteins, characterized by specific features, which form a signature of extreme multifunctionality. Overall, extreme multifunctional proteins are enriched in linear motifs and less intrinsically disordered than network hubs. We also provide MoonDB, a database containing information on all the candidates identified in the analysis and a set of manually curated human moonlighting proteins.

## Abstract (web summary)

Proteins are sometimes implicated in separate and seemingly unrelated processes, so called moonlighting functions. Here the authors use bioinformatics tools to identify extreme multifunctional proteins and define a signature of extreme multifunctionality.

## Graphical abstract

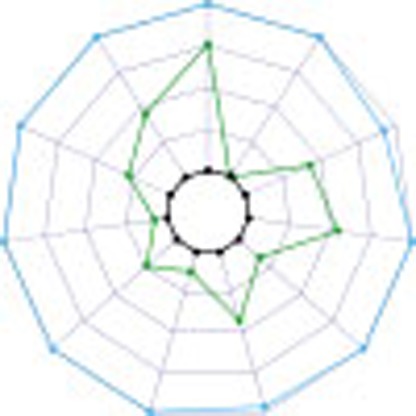

How common are multifunctional proteins? Is protein multifunctionality the exception or the rule? Is there a scale of multifunctionality? These questions are particularly relevant in the context of the C-value paradox, the fact that genome size does not correlate with organismal complexity^1^. Protein multifunctionality may be one of the ways a cell makes more with less.

Like gene/protein function^2^, gene/protein multifunctionality can be defined at each of the different organizational levels, ranging from molecular to organismal. At the molecular level, the catalytic promiscuity of a single domain, that is, its ability to catalyse both a primary substrate-specific function and a different, secondary reaction, can account for multifunctionality. So does the presence of multiple different catalytic domains within the same protein^3^. At the cellular level, multifunctionality corresponds to gene products involved in multiple biological processes (BPs), often revealing regulatory roles. Finally, at the organismal level, pleiotropy is detected when changes in a single locus lead to multiple phenotypic effects.

Although protein multifunctionality is widely recognized and certain multifunctional proteins have been extensively studied (for example, TP53), multifunctional proteins as a *group* have not received much attention in the literature. As a result, multifunctional proteins suffer from a lack of characterization, unclear definitions and have only been studied individually, on a case by case basis. This may be partly due to the lingering effects of the one gene, one enzyme and by extension, one function hypothesis^4^. As proteins tend to be studied in terms of their known function, alternate functions—often serendipitously discovered—are arbitrarily relegated to secondary status.

The past few years have seen a growing interest in moonlighting proteins (MPs), a special sub-class of multifunctional proteins^3^,^5^. They are defined as ‘special multifunctional proteins, because they perform multiple autonomous, often unrelated, functions without partitioning these functions into different protein domains'^6^. The human aconitase is an oft-cited example: an enzyme of the tricarboxylic acid cycle that turns into a translational regulator when the iron concentration changes^7^; as is hyaluronan-mediated motility receptor (HMMR), a nuclear microtubule-associated protein that, in certain cancers, is exported to the extracellular matrix where it binds CD44, ultimately promoting metastasis (reviewed in refs 8, 9).

MPs may play important roles in normal or pathological contexts and their study could improve our understanding of complex genotype–phenotype relationships. However, owing to the serendipitous nature of their discovery, the number of proteins explicitly described as MPs is still very low, impairing our ability to define common characteristics on which a systematic search can be based. Consequently, there has been no large-scale attempt to identify them. In addition, although sequence analysis tools and computational domain predictions are very useful in establishing a protein's molecular functions, they rarely predict the cellular or physiological functions, and are therefore ill-suited for the identification of MPs. Furthermore, multifunctionality often blurs the possible functional inferences made from sequence similarity searches^10^ and current algorithms rarely identify additional functions with a high confidence score^11^, hindering conclusive predictions. Finally, that known MPs can switch between functions upon change of (i) subcellular localization, (ii) physicochemical environment, (iii) oligomeric state, (iv) interacting partners or a combination thereof^12^ means that predicting alternate functions should go beyond sequence analysis.

Protein–protein interaction (PPI) networks (interactomes) highlight the modularity of cellular processes and allow deciphering protein functions at the cellular level. PPI networks represent the set of all detected interactions between each of their constituent proteins in a time and cell-type independent manner. All possible interactions of a given protein are thus shown simultaneously in the same network, therefore enabling the identification of proteins involved in different processes in different contexts, a prerequisite for the study of MPs. Indeed, MPs are expected to specifically interact with different sets of protein partners, either simultaneously or not, depending on the function performed.

We therefore reasoned that, as a first step towards the large-scale identification of MPs, PPI networks can be used to identify proteins whose multiple functions are very dissimilar to one another. Although these ‘extreme multifunctional proteins' (EMFs) will not all adhere to the strict definition of MPs, detecting this form of multifunctionality is interesting in itself and, in addition, we can expect ‘classical' MPs to be a subset of EMFs.

By associating network topological information with existing protein annotations, we have identified 430 such EMFs in the human interactome. We show that they form a distinct sub-group of proteins in the human network, characterized by specific features, which set them apart from other multifunctional proteins and which, when combined, form a signature of extreme multifunctionality. We also present MoonDB, a database containing diverse information on our set of 39 manually curated human ‘known MPs' and on all the candidates identified in this analysis. Finally, we discuss the relationship between extreme multifunctionality and moonlighting in light of our results.

## Results

### Principle of inference for EMFs

Multifunctional proteins are expected to perform their different functions through different interaction partners. We therefore need to identify proteins at the intersection of sets of functionally related proteins. First, overlapping protein sets were identified in the human interactome using overlapping cluster generator (OCG)^13^, an algorithm that covers a network with a system of overlapping clusters. These clusters are formed by highly interconnected proteins, which tend to be involved in the same cellular processes and may include protein complexes. We chose OCG because, as we have previously demonstrated^13^, it is particularly well suited for the detection of multifunctional proteins and it fares better than other algorithms on sparse graphs such as PPI networks. Second, the cellular process(es) in which the clusters are involved were identified based on the BP Gene Ontology (GO)^14^ annotations of their constituent proteins: GO terms annotating at least 50% of a cluster's proteins are assigned to the cluster, which can now be called a ‘functional module'. Each individual protein then inherits the annotations of its module(s) in addition to its own. This annotation procedure favours the detection of functionally homogeneous clusters—likelier to represent functional modules—and maximize the number of clusters successfully annotated. Finally, to distinguish extreme from ‘classical' multifunctional proteins, proteins found at the intersection of functional modules annotated to *dissimilar functions* were identified.

**Figure 1:**
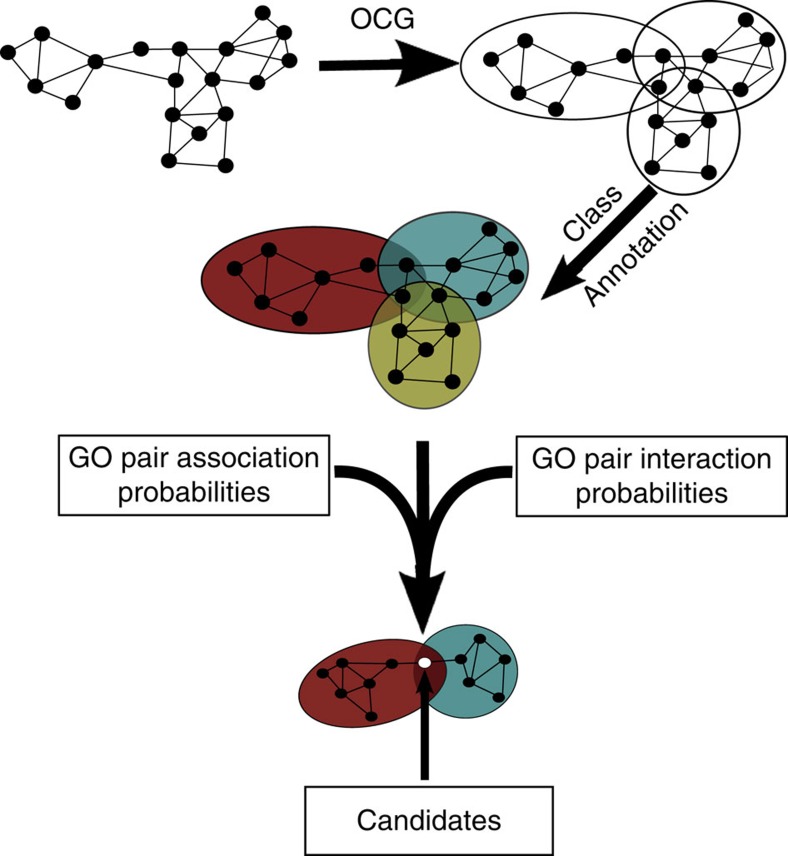
MoonGO: the EMF identification pipeline. Overlapping clusters are extracted from a PPI network using OCG. Clusters are annotated according to the GO annotations of their constituent proteins. Potential EMFs are then identified at the intersection of clusters involved in unrelated biological processes according to PrOnto GO term association probabilities.

Function (dis)similarity is given by two metrics of GO term association (made available in the PrOnto database; see Methods and (http://tagc.univ-mrs.fr/pronto)) based on the frequency of co-occurrence of a GO term pair either in a protein's or in a pair of interacting partner's annotations. Using these metrics ensures that the multiple functions in which the candidate protein is found to be involved are very rarely performed (i) by a single protein and (ii) by interacting proteins, two proxies that we consider indicators of *unrelated functions*. Our pipeline (MoonGO) is explained in more detail in the Methods section and is summarized in Fig. 1.

### A set of 430 EMF candidates

We have applied our pipeline to a large, high-quality human interactome (74,388 interactions between 12,865 nodes, Supplementary Data 1) built by extracting data from online databases (see Methods). The 855 overlapping clusters returned by OCG contained 33.4 proteins on average. Of these, one or several BPs were assigned to 633 (74%), based on the annotation of their constituent proteins (Supplementary Data 2). All network proteins belonged to at least one annotated cluster. As expected from a previous analysis, around a third of the interactome (3,846 proteins, 29%) belonged to several clusters and can therefore be considered multifunctional^13^. Of these, 430 proteins (10%) are found at the intersection of clusters annotated to dissimilar functions and are considered EMF candidates (Supplementary Data 3).

As candidates are defined with respect to their module's annotations, their identification is dependent on the quality of these annotations. Said quality was assessed by performing three types of randomization tests. First, the annotations of all proteins were shuffled, and candidates were identified using these randomized annotations. Over 100 trials, on average only 104.78 of 855 clusters were annotated compared with 633 for the real data, demonstrating that such high number of functionally homogeneous clusters cannot be found by chance. Consequently, the number of identified candidates markedly decreased in these conditions (7.55 versus 430 for the real data). Second, the network topology was randomized by drawing random edges between the nodes. As expected, since this destroys the modular structure of the network, virtually no candidates were found. The process was repeated ten times and only 0.4 candidates were identified on average, clearly showing that our results cannot be found in a random network. Third, the topology was randomized while keeping the same degree distribution. In other words, the number of nodes with a given degree was the same but which node had which degree was randomized. The process was repeated ten times and our pipeline was applied to these randomized networks. As expected, we found almost no results: only 26.8 EMF candidates were identified on average.

Candidate identification also depends on the annotation probabilities establishing the functional dissimilarity of modules. We therefore recalculated the number of candidates when reshuffling the probabilities of association between GO term pairs. An average of 1.03 candidates were identified over 100 runs, once more indicating that our results cannot be obtained by chance.

The validity of the functional module approach was verified by demonstrating that the GO terms that led to the candidates' discovery are not among their existing annotations but were brought in by the annotation inheritance process depending on their module membership. Indeed, only 71 candidates (17%) were already annotated to both terms used to identify them, 209 (51%) were annotated to one term and inherited the other from their modules' annotations, whereas 128 (31%) were not annotated to either term, therefore inheriting both terms from their modules. This further confirmed the power of interaction network analyses for function prediction.

Finally, the quality of the inferred annotations was assessed using a leave-one-out approach. For each of the clusters identified, we removed one of its proteins' annotations, annotate the cluster and infer the cluster's annotations to the protein. Doing so, we assign at least one of the known annotations correctly in 62.6% of cases, indicating that our approach is capable of rescuing known protein annotations, therefore suggesting that the novel annotations we infer are trustworthy.

As MPs are a subset of EMFs, we compiled a list of 39 known human MPs from the literature and checked whether they were found as candidates. Six of the thirty-nine belonged to dissimilar modules and were found. Although finding 6 out of 39 when identifying 430 candidates out of 12,865 represents an enrichment of 4.6-fold compared with expected, with a significant *P*-value (1.4e^−3^, hypergeometric), 6 of the remaining 33 could not be found by MoonGO since they belonged to clusters that could not be annotated. We cannot thus exclude that the remaining 27 proteins were missed because of ill-annotated clusters or because all their interactions have not been discovered yet. Overall, these different assessments confirmed the specificity of our approach and consolidated our confidence in the identified candidates.

The candidates were found linking 141 different pairwise function combinations between 55 different GO terms. These annotation pairs are functionally dissimilar by both annotation and interaction probabilities (Supplementary Data 4). Most of the candidates (>90%) were found annotated to dissimilar functions involving on the one hand nucleic acid-linked metabolic processes and on the other (i) signalling activity, (ii) localization or (iii) transport (see Table 1 and Supplementary Data 4).

### Candidate characterization

To investigate whether EMF candidates (Cands) form a distinct group of proteins with respect to the other proteins of the network, the candidates were analysed to identify common trends and features that characterize them (Table 2). For each characteristic studied, they were compared with several categories: (i) the entire network's proteins, (ii) proteins that belong to multiple clusters but are not candidates as those clusters are not annotated to significantly different GO terms (Multi-non-candidate (NC)), (iii) all multiclustered proteins (Multi), (iv) proteins belonging to a single cluster (Mono), (v) all NC proteins and (vi) network hubs, defined here as those nodes whose degree is at least twice the network average (⩾25). Note that these categories are not all mutually exclusive. Since the candidates are defined as those proteins found at the intersection of modules annotated to dissimilar terms, they are all, by definition, multiclustered. Therefore, to identify characteristics common to EMFs that distinguish them from other multifunctional proteins, features shared by candidates but not by the Multi-NCs need to be found.

### Candidate network topological features

**Figure 2:**
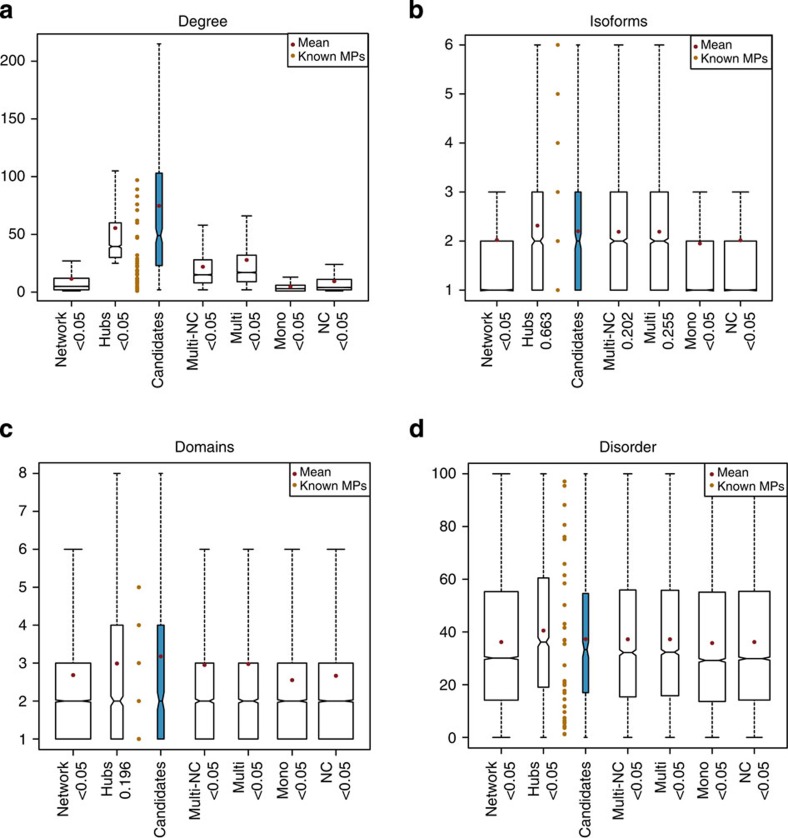
Protein features. (**a**) Protein degree. (**b**) Protein isoforms. (**c**) The number of Pfam domains (including PfamB) predicted on each protein. (**d**) Protein disorder as calculated by disopred. The numbers shown are the percentage of a protein's residues that are disordered. Outliers are not shown. Red dots indicate mean values and the camel dots between Candidates and Hubs are the values of the known moonlighting proteins.

Candidates have a significantly higher degree than the Multi-NCs. On average, they interact with 74.6 proteins compared with 21.9 for the Multi-NCs (Wilcoxon test *P*-value=1.27e-96; Fig. 2a). They consequently belong to more network clusters (mean Cands (15.2) versus Multi-NCs (3.8), Wilcoxon test *P*-value=1.15e-150; Supplementary Fig. 1) and are significantly more central to the network than Multi-NCs (mean Cands (235,005.6) versus Multi-NCs (46,177.2), Wilcoxon test *P*-value=1.44e-112) as shown by their betweeness scores (Supplementary Fig. 2), a measure of how central a given node is in the network, calculated by quantifying the number of times a node acts as a bridge along the shortest path between two other nodes. Not surprisingly, candidates are also more connected to each other according to a shortest path analysis than the Multi-NCs (Supplementary Fig. 3).

Note that although candidates tend to be more connected than hubs (mean Cands (74.6) versus Hubs (55.5)) not all candidates have a high degree (see Fig. 2a) and only 20% of network hubs were found as candidates, demonstrating that a high degree is neither necessary nor sufficient for consideration as a candidate.

### Candidate sequence and structural features

Protein interaction and functional annotation databases tend to refer to genes rather than gene products and do not differentiate between different protein isoforms^15^. It is therefore possible that the multiple functions of our candidates are actually carried out by different isoforms of the same gene. However, candidates do not have significantly more isoforms than the Multi-NCs (mean Cands (2.2) versus Multi-NCs (2.1), Wilcoxon test *P*-value=0.2; Fig. 2b), suggesting that the number of isoforms is not a defining characteristic. As expected for highly multifunctional proteins, candidates have more domains than the Multi-NCs (mean Candidates (3.2) versus Multi-NCs (3.0), Wilcoxon test *P*-value=0.0001, Fig. 2c). They are not, however, significantly longer (mean Cands (664.7) versus Multi-NCs (614.4), Wilcoxon test *P*-value=0.09, Supplementary Fig. 4).

The candidates were also, like the hubs, more conserved than the Multi-NCs (mean Cands (43.7) versus Multi-NCs (42.6), Wilcoxon test *P*-value=0.007; see Methods and Supplementary Fig. 5).

As structural disorder can allow conformational changes, we used DISOPRED^16^ to predict disordered residues and analysed both the percentage of disordered residues per protein (Fig. 2d) and the number of stretches of consecutive disordered residues of different lengths (Supplementary Fig. 6). Interestingly, although candidates are not distinguishable from either Multi-NCs or the network average, they are significantly less disordered than hubs (mean Cands (37.3) versus Hubs (40.5), Wilcoxon test *P*-value=0.028, see Fig. 2d). This trend was confirmed by the results of ten other disorder predictors whose results were retrieved from the D2P2 database^17^ (see Supplementary Figs. 7–16). These results suggest that, despite their high average degree, EMFs, unlike regular hubs, are under stronger selective pressure to maintain a stable secondary structure.

**Figure 3:**
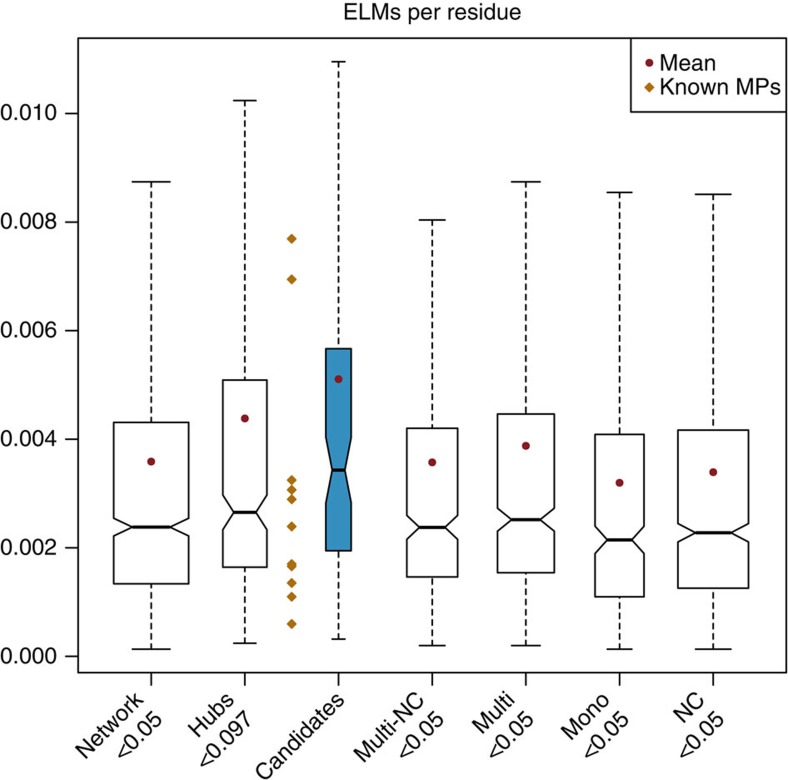
ELMs per residue. The plots show the number of ELMs divided by the length of each protein.

Eukaryotic linear motifs (ELMs) are short stretches of amino acids often located within intrinsically disordered regions, which have been shown to help the targeting of proteins to specific subcellular localizations, determining the modification state of proteins, or regulating protein activity in a context-dependent manner^18^. We checked their numbers in proteins of each group, particularly in their disordered regions. Interestingly, candidates are enriched in ELMs per residue compared with the Multi-NCs (mean Cands (0.0051) versus Multi-NCs (0.0035), Wilcoxon test *P*-value=3e-4, Fig. 3), a trend which is more pronounced when considering only ELMS that fall within disordered regions (mean Cands (0.009) versus Multi-NCs (0.006), Wilcoxon test *P*-value=8e-4, Supplementary Fig. 17). Notably, ELMs are more enriched among disordered residues in candidates (1.8-fold, mean per disordered residue (0.009) versus per residue overall (0.005), Wilcoxon test *P*-value=0.03) than in Multi-NCs (1.5-fold, mean per disordered residue (0.006) versus per residue overall (0.004), Wilcoxon test *P*-value=0.29). These results indicate that candidates contain more ELMs, particularly in disordered regions. With respect to hubs, the same trend was observed, although not statistically significant. It therefore appears that candidates differ from hubs in their disorder content and from Multi-NCs in the number of linear motifs per residue.

Finally, the different groups were also checked for the presence of proteins identified as containing ELMs involved in functional switches, the status of which ultimately affects the function of the ELM-containing protein (collected in the switchELM database^19^). Candidates show a 6.2-fold enrichment in such proteins (hypergeometric *P*-value=1.2e-27), compared with a 1.8-fold among Multi-NCs (*P*-value=2.44e-14), 4.68-fold for hubs (*P*-value=1.07e-62) and a depletion in Mono (2.7-fold less, *P*-value=2e-49). When different types of functional switches are considered, the candidates are enriched in proteins containing binary switch motifs (with an ON/OFF state) modulated by allosteric effects (1.6-fold, *P*-value=4.85e-2), compared with Multi-NCs and hubs, which show no enrichment. As numbers are low (27 such proteins in the interactome, 9 of which are EMF candidates, *P*-value=4.85e-2), we cannot reasonably extrapolate this observation to the complete EMF data set. However, this particular finding combined with the higher occurrence of ELMs in the candidates reinforces their functional significance as extreme multifunctional and potential MPs.

### Candidate annotations and expression

The candidates have significantly more BP annotations (mean=16.8, Supplementary Fig. 18) than both hubs (mean=13.0, Wilcoxon test *P*-value=0.00014) and Multi-NCs (mean=9.3, Wilcoxon test *P*-value=1.56e-21), as expected for proteins involved in multiple functions. This is not introducing a bias in the analysis as only 17% of the candidates were already annotated to the dissimilar GO pairs used to identify them as candidates (see ‘A set of 430 EMF candidates'). Finally, the candidates are more ubiquitously expressed at the mRNA level (mean=24.8 tissues, Supplementary Fig. 19) than Multi-NCs (mean=19.8 tissues, Wilcoxon test *P*-value=5.53e-05), raising the possibility that their different functions could be performed in different tissues.

### Candidate involvement in disease

Multifunctional proteins in general and EMFs in particular are expected to be involved in disease since impairing their function can affect multiple cellular processes. We therefore used online mendelian inheritance in man (OMIM)^20^ annotations to test our candidate's involvement in human diseases and found a total of 113 out of 430 candidates associated with 229 different diseases.

There was a 7.6-fold overrepresentation of disease-associated proteins among candidates (hypergeometric *P*-value=2.1e-07), but only a 6.2-fold and 5.8-fold in all Multis (hypergeometric *P*-value=9.3e-19) and hubs (hypergeometric *P*-value=0.0003), respectively. As proteins that are involved in multiple processes are more likely to cause disease when perturbed, these results support our claim that multi-clustered proteins are likely multifunctional and reinforce the differences between candidates and hubs.

We also used the list of 435 cancer genes from ref. 21 to check for overrepresentation of cancer-associated genes among candidates. Once more, we found that those are clearly overrepresented (3.8-fold, hypergeometric *P*-value=6.8e-2) among the candidates, whereas their overrepresentation among all Multis (twofold, hypergeometric *P*-value=2.7e-38) and hubs (threefold, hypergeometric *P*-value=2.8e-43) is less important. This suggests that although all multi-clustered protein groups are enriched in cancer genes, candidates are once more different from other multi-clustered proteins and hubs.

### A signature of EMFs

The signature was built by combining all tested features that displayed a significant statistical difference between the candidates and the Multi-NCs on the one hand and the hubs on the other.

**Figure 4:**
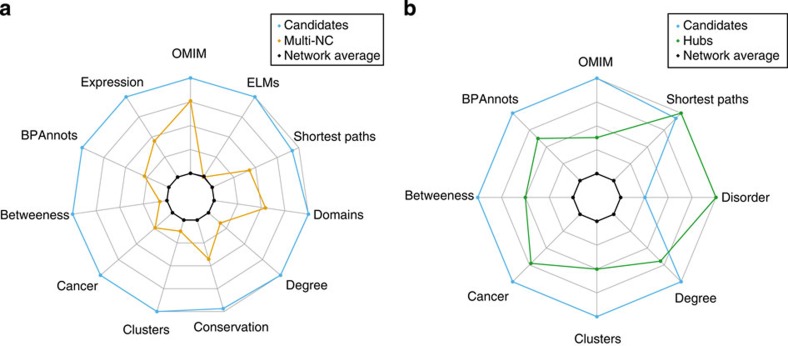
Radar plots. Radar plots showing the characteristics that were significantly different in candidates with respect to Multi-NCs (**a**) and hubs (**b**). Mean values are plotted for all features except association with cancer, where fold overrepresentation is shown. For Shortest Paths, the outermost data point is the *most* connected, that is, has the *smallest* Shortest Path value. For all others, the outermost data point is the one with the greatest value. These plots are purely descriptive and were built once the candidates were found. The features they describe were not used to identify the candidates.

Our analyses therefore describe a first set of characteristics (summarized in Fig. 4) of EMFs that differentiates them from other multi-functional proteins. They tend to have more interactors, to belong to more clusters, to be more central and more connected to each other in the network; they also have more annotations, more domains are more conserved and contain more linear motifs. They have a greater tendency to be involved in disease and tend to be expressed more ubiquitously.

Another set of characteristics was defined with respect to hubs. Candidates tend to have more interactors, to be more central to the network although less connected to each other and to belong to a greater number of network clusters. They are more likely to be involved in disease and have more BP annotations. Very interestingly, they tend to be less disordered than hubs, with the same average disorder as the network.

To ensure that these signatures are not influenced by highly studied proteins (such proteins often have an artificially high degree in PPI networks because their interactions have been exhaustively characterized), we repeated the analysis on a smaller human PPI network built exclusively from large-scale yeast two-hybrid data (CCSB network^22^). Despite the much smaller size of this network (15,617 interactions between 4,494 proteins), we could still observe the same global trends in the 43 candidates found by our pipeline for most of the features of the signatures (Supplementary Figs 20–31). Although the low number of candidates kept most of these observations below the significance threshold, it is interesting to note that the 43 candidates were still significantly less disordered than the hubs (mean Cands (36.0) versus hubs (47.5), Wilcoxon test *P*-value=0.01). As these results were obtained in a bias-free network, they reinforce the robustness of our findings on the large interactome.

### Example candidates

Although a discussion of each of our candidates is clearly beyond the scope of a single paper, we highlight a few particularly interesting cases here. Note that the cluster's Cellular Component (CC) annotations shown below are only indicative and were not used in the prediction of candidates.

Receptor tyrosine-protein kinase erbB-2 (ERBB2) is a member of the epidermal growth factor receptor family and an essential component of the neuregulin-receptor complex, which regulates outgrowth and stabilization of peripheral microtubules^23^. Besides its signalling role, ERBB2 is also a transcription factor involved in the transcription of rRNA genes by RNA Pol I (ref. 24). We find ERBB2 at the intersection of two clusters, one annotated to ‘cellular nitrogen compound metabolic process' (BP, GO:0034641), a parent term of ‘transcription, DNA-templated', and to ‘cytosol' and ‘nucleus' (CC), the other annotated to ‘signal transduction' (BP, GO:0007165) and ‘plasma membrane' (CC). Our method, therefore, correctly identified ERRB2 as an EMF and assigned it to its different and unrelated real functions.

Protein RPP40, a component of the nuclear ribonuclease P, known to cleave the 5' end of tRNA molecules during their processing, was found at the intersection of a cluster annotated to ‘cellular nitrogen compound metabolic process' (BP, GO:0034641)—a parent term of its *bona fide* annotation ‘tRNA processing'—and ‘nucleus' (CC), and another annotated to ‘signal transduction' (BP, GO:0007165) and ‘plasma membrane', and ‘cytosol' (CC), suggesting a possible signaling role for this nuclear protein. Interestingly, the mitochondrial counterpart of RNase P assumes the same function, although formed by three proteins not related at the sequence level to the nuclear form. It has been proposed that this mtRNase P complex ‘was not built simply from components of a preexisting nucleolytic pathway but by combining components from different, essentially unrelated biochemical pathways'^25^, which would strongly suggest possible moonlighting functions for the proteins dedicated to this cellular process.

WBP4 is a spliceosome-associated protein, which promotes pre-mRNA splicing. It is found at the intersection of a cluster annotated to ‘RNA splicing, via transesterification reactions with bulged adenosine as nucleophile' (BP, GO:0000377) and ‘nucleoplasm' (CC), which would correspond to its known function, and another annotated to ‘response to endogenous stimulus' (BP, GO:0009719) and ‘cytosol',‘nucleoplasm part' and ‘intracellular non-membrane-bounded organelle' (CC), suggesting a novel role for this protein. Although no involvement in a signalling pathway has been shown to date, the protein contains two WW domains able to interact with proline- or phosphoserine-phosphothreonine-containing motifs and known to mediate regulatory interactions in various signalling pathways such as Hippo^26^.

### MoonDB

We have collected our results in MoonDB, a database that includes the 39 human MPs used here as well as the full list of EMF candidates. For all proteins, MoonDB provides easy access to diverse information (sequence, domain organization, functional annotations, involvement in disease and so on). In addition, for the candidates identified here, information about the functional modules from the graph and the functional dissimilarity of the GO pairs, which allowed their identification, are provided. MoonDB is available at http://tagc.univ-mrs.fr/MoonDB.

## Discussion

What are MPs except proteins whose different functions have been serendipitously discovered and whose current definition has been molded to fit them? Do MPs really form a protein class unto themselves, with their own as yet undiscovered characteristics? Are they proteins that play truly different roles in the cell, or do we consider these roles different because we have not discovered their functional links yet?

In this context, we have chosen to avoid the term *moonlighting* here, largely because the current definition is too stringent. According to the primary definition^5^, a multifunctional protein must not partition its functions into different domains to be considered moonlighting. However, it is very likely that when no second domain is identified *in silico*, it is simply because the domain in question is either unknown or below detection thresholds. To establish that a protein's two functions are performed by the same domain would therefore require experimental analyses. Human protein XRRC5, for example, was known to be involved in DNA repair yet was found to interact with metalloproteinases of the extracellular matrix^27^. It was only after this discovery that the protein was shown to contain a vWF domain whose sequence had diverged to the point that it was below the detection thresholds. We have, therefore, coined the term EMFs to describe proteins whose multiple functions are very different to one another. We feel that such proteins are of interest irrespective of whether they split their functions across multiple domains or whether their functions are independent. In addition, some multifunctional proteins are not considered moonlighting simply because their alternate functions are very well known. TP53, for example, fits the definition of MPs yet has never been considered as one. This is just one of many such ‘sunlighting' proteins, which, were they to be discovered today, would be classed as moonlighting. EMFs include both classes of protein.

We have previously shown that using algorithms like OCG that is able to cluster proteins into multiple graph modules allows the identification of multifunctional proteins^13^. These modules correspond to the functional units of the network, are composed of groups of highly connected proteins involved in the same cellular function^28^ and permit function prediction when containing uncharacterized proteins^29^,^30^. In the present work, we have used the functional module approach to identify those multifunctional proteins that exhibit extreme multifunctionality. This was made possible by the tailored development of the MoonGO pipeline, which uses an original function dissimilarity measurement, the PrOnto probabilities. The latter are based on the frequency of co-occurrence of GO term pairs in protein annotations or among the annotations of interacting protein pairs and are specific to the species being studied. These probabilities reflect biases towards infrequent association implying functional dissimilarity or, conversely, frequent association indicating functional similarity. Finally, although there have long been calls for PPI network analyses for the identification of highly multifunctional and MPs^31^, such an extensive and global approach has never been undertaken. We show here that EMF candidates can be identified without *a priori* by the combination of interaction data analysis and the processing of current knowledge on protein function such as GO annotations.

Our method identified 3.3% of the human interactome (430 of 12,865 proteins) as candidate EMFs. Although an estimation of the expected proportion of EMFs is rather difficult, it is of note that our candidates from the bias-free CCSB network represent a similar proportion (1%), another argument confirming the significance of our results.

We have used the different characteristics shared by this group of proteins to define a signature of extreme multifunctionality, which distinguishes them from other network proteins. Importantly, this signature is reinforced by the fact that the same trends were observable in the candidates identified in the much smaller CCSB network, which is free of the biases associated with highly studied proteins.

Although EMFs are in many ways similar to hubs (degree of at least twice the network average, ⩾25) and have many of the same characteristics, not all EMFs are hubs. It is particularly interesting that EMFs tend to be less disordered than hubs given that intrinsic protein disorder can enable proteins to adopt different conformations that can assist EMFs in their multiple functions (as already suggested for MPs^32^). Furthermore, although intrinsic disorder has been shown to be important for hub's multiple interactions (for example, see refs 33, 34), candidates are significantly less disordered than hubs (see Fig. 2d) despite 361 of them (88%) being hubs. Disorder therefore appears to be important only for a specific subset of hubs, those which are not EMFs. That EMFs are just as disordered as the network average extends the observation made by Hernandez *et al*.^35^ on a small number of known MPs to the broader class of EMFs.

So, what makes EMFs special compared with other multifunctional proteins? In terms of network topology, a typical EMF is likely to have a higher degree, to belong to more network modules and to be more central to the network. It is more likely to be involved in multiple diseases and to be expressed more ubiquitously, suggesting that it can perform different functions in different tissues. It will also have more domains, be more conserved than a classical multifunctional protein, and contain more short linear motifs (ELMs). These ELMs are short conserved sequences mostly located in disordered regions. They form low-affinity interaction interfaces, are involved in transient interactions and, importantly, mediate molecular decision-making in cell regulation^19^,^36^,^37^. That ELMs (i) can bind competitively or sequentially to different interaction partners in a context-dependant manner, (ii) provide a large panoply of conditional regulatory types through interactions^19^ and (iii) are more numerous in EMFs, provides a possible molecular explanation of the functional versatility of these proteins. This clearly calls for further studies.

Overall, the signatures we defined clearly show that EMFs form a distinct subgroup of multifunctional proteins exhibiting characteristics that distinguish them from hubs, classical multifunctional proteins and the network in general and can pave the way towards a better understanding of protein moonlighting.

## Methods

### Networks

Interaction data were retrieved using the PSIQUIC^38^ interfaces of the APID^39^, BioGrid^40^, IntAct^41^, DIP^42^, MINT^43^, MatrixDB^44^, Reactome^45^, InnateDB^46^, MolCon, Spike^47^ and TopFind^48^ databases. They were filtered according to their identification methods and only binary interactions between proteins were kept. Protein names were mapped to UniProt IDs, and sequences clustered using CD-HIT^49^. TrEMBL/SwissProt protein pairs sharing ⩾95% similarity were considered to be the same protein: interactions of the TrEMBL protein were then inherited by the Swiss-Prot protein. Self interactions were discarded. The final result was high-quality interactomes consisting entirely of experimentally verified, direct, binary interaction pairs.

The CCSB network was downloaded from the CCSB Human Interactome database^22^.

### Cluster Identification and annotation

Clusters were generated using OCG^13^ and default options. The clusters were annotated according to the BP GO annotations of its constituent proteins. A cluster will be annotated to a GO term *iff* ⩾50% of annotated proteins in that cluster share that GO term and all member proteins will inherit the annotation(s) of the cluster. Both direct GO annotations and all parent terms are taken into account. Note that those clusters that can only be annotated to the root of the ontology (that being the only term shared by ⩾50% of their constituent proteins) are given the annotation ‘BP unknown'. Because the quality of computationally inferred GO annotations has now been shown to rival that of curated non-experimental annotations^50^, we use all BP GO annotations in this study, irrespective of their evidence codes.

### Function association probabilities

We have developed two measures of GO term functional similarity, one (the annotation probabilities) measures whether two GO terms are found annotating the same protein more often than expected by chance. The second (interaction probabilities) measures whether there are more interactions between proteins annotated to GO term X and proteins annotated to GO term Y than would be expected by chance.

For both metrics, we have calculated the probability of association between two GO terms *GO*_1_ and *GO*_2_ using the hypergeometric distribution 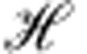 (*N*, *K*, *n*, *k*), where, for the annotation probabilities, *N* is the number of proteins with at least two different direct annotations, *K* is the number of proteins directly annotated to *GO*_1_, *n* is the number of proteins annotated to *GO*_2_ and *k* is the number of proteins annotated to both terms.

For the interaction probabilities, *N* is the number of interactions in our network between proteins with at least two different annotations, *K* is the number of interactions involving proteins annotated to *GO*_1_, *n* the number of interactions involving proteins annotated to *GO*_2_ and *k* the the number of interactions between a protein annotated to *GO*_1_ and one annotated to *GO*_2_. To be considered dissimilar by our pipeline, two GO terms must have a probability of ≤0.05 for both metrics.

We have made these probabilities available in the PrOnto database, which is freely available at http://tagc.univ-mrs.fr/pronto/. Note that although PrOnto offers cross-ontology probabilities, only the BP probabilities were used in the present work to identify candidates.

A more detailed explanation of our choice of developing PrOnto rather than using existing semantic similarity measures and a comparision of PrOnto and Semantic Similarity measures are provided as a Supplementary Note 1.

### MoonGO

MoonGO, our EMF prediction tool, uses the annotated clusters and PrOnto probabilities to search the network for proteins found connecting clusters annotated to dissimilar BP GOs. It then looks for nodes that are members of both clusters (see Fig. 1)

As the number of GO term pairs analysed is very large (106618), MoonGO will correct for multiple testing by multiplying the *P*-value of association between two terms by the number of tests performed to obtain a corrected e-value. MoonGO uses both the annotation and the interaction probabilities to identify its candidates. A candidate is kept if both the annotation and interaction e-values of the BP GO term pairs associated with it are significant. For the work presented here, we have used an e-value significance threshold of ≤0.05.

### Analyses

The betweeness and shortest path analyses were done using the R igraph library^51^. Domain predictions were made using pfam_scan.pl^52^, the results shown in Fig. 2c include both PfamA and PfamB domain predictions. Protein disorder values were obtained using disopred^16^ with default settings. Figure 2d was generated by plotting the sum of disordered residues of each protein. Protein isoform information and OMIM annotations were downloaded from UniProt and protein annotations from the EBI's QuickGO server (https://www.ebi.ac.uk/QuickGO/). Expression data were taken from ref. 53. Protein phosphorylation predictions were made using GPS 2.1 (ref. 54).

### Conservation

The protein sequences of all network proteins and their annotated homologues in yeast, mouse, fly and worm were aligned against each other using t_coffee^55^. The homologous sequences were taken from EnsEMBL^56^. To obtain an indicative conservation value, the bit score of the alignments was divided against the length of the human homologue and the resulting values were compared.

### ELMs

The coordinates of all annotated ELMs in our network's protein were obtained from the ELM database^57^. These were mapped to the disordered regions predicted by DISOPRED. Only ELMs that fell entirely within or which overlapped perfectly with a disordered region were counted. ELMs that overlapped but extended beyond the disordered region were ignored. To avoid misrepresentations caused by differing protein or disordered region lengths, the number of ELMs in disordered regions was divided by the total number of disordered residues of each protein. Similarly, the number of ELMs per protein was divided by the total number of residues of each protein.

### Known MPs and MoonDB database

The MoonDB database was written using a combination of HTML 4.01, PHP 5.5 and Javascript, the data are stored in a MySQL 5.5 database. We have compiled a manually curated list of *bona fide* human MPs, which we have made available, along with our own predictions, at the MoonDB database.

## Additional information

**How to cite this article:** Chapple, C. E. *et al*. Extreme multifunctional proteins identified from a human protein interaction network. *Nat. Commun.* 6:7412 doi: 10.1038/ncomms8412 (2015).

## Supplementary Material

Supplementary Information Supplementary Figures 1-32 and Supplementary Note 1

Supplementary Data 1 The human interactome used in this study

Supplementary Data 2 The annotated OCG class file used in this study

Supplementary Data 3 List of EMF candidates identified in this study

Supplementary Data 4 The list of GO term pairs used to identify the candidates

## Figures and Tables

**Table 1:** Dissimilar biological processes.

**%**	**Dissimilar biological processes**	
53.5	Nitrogen or nucleic acid metabolism	Signalling
7.3	Nucleic acid metabolism	Localization
6.8	Macromolecular metabolic process	Transport
5.9	RNA metabolic process	Signalling
4.7	Nucleic acid metabolism	Transport
4.5	Gene expression	Transport
3.6	Macromolecular metabolic process	Localization
2.9	Nucleic acid metabolism	Physiological processes
2.7	Nucleic acid metabolism	Phosphorus metabolism
1.2	Nucleic acid metabolism	Development

Top 10 dissimilar function pairs by the percentage of candidates identified.

**Table 2:** Candidate features.

## References

[b1] Doolittle W. F. Is junk dna bunk? a critique of encode. Proc. Natl Acad. Sci. USA 110, 5294–5300 (2013). 23479647 10.1073/pnas.1221376110 PMC3619371

[b2] Jacq B. Protein function from the perspective of molecular interactions and genetic networks. Brief. Bioinform. 2, 38–50 (2001). 11465061 10.1093/bib/2.1.38

[b3] Copley S. D. Moonlighting is mainstream: paradigm adjustment required. Bioessays 34, 578–588 (2012). 22696112 10.1002/bies.201100191

[b4] Tatum E. L. & Beadle G. W. Genetic control of biochemical reactions in neurospora: an ‘aminobenzoicless' mutant. Proc. Natl Acad. Sci. USA 234–243 (1942). 16578042 10.1073/pnas.28.6.234 PMC1078456

[b5] Jeffery C. J. Moonlighting proteins. Trends Biochem. Sci. 24, 8–11 (1999). 10087914 10.1016/s0968-0004(98)01335-8

[b6] Huberts D. H. E. W., Venselaar H., Vriend G., Veenhuis M. & van der Klei I. J. The moonlighting function of pyruvate carboxylase resides in the non-catalytic end of the tim barrel. Biochim. Biophys. Acta 1803, 1038–1042 (2010). 20359504 10.1016/j.bbamcr.2010.03.018

[b7] Volz K. The functional duality of iron regulatory protein 1. Curr. Opin. Struct. Biol. 18, 106–111 (2008). 18261896 10.1016/j.sbi.2007.12.010 PMC2374851

[b8] Maxwell C. A., McCarthy J. & Turley E. Cell-surface and mitotic-spindle rhamm: moonlighting or dual oncogenic functions? J. Cell Sci. 121, 925–932 (2008). 18354082 10.1242/jcs.022038

[b9] Jiang J. . Multifunctional proteins bridge mitosis with motility and cancer with inflammation and arthritis. Sci. World J. 10, 1244–1257 (2010). 10.1100/tsw.2010.141 PMC5763930 20602082

[b10] Gómez A., Domedel N., Cedano J., Piñol J. & Querol E. Do current sequence analysis algorithms disclose multifunctional (moonlighting) proteins? Bioinformatics 19, 895–896 (2003). 12724303 10.1093/bioinformatics/btg111

[b11] Khan I., Chitale M., Rayon C. & Kihara D. Evaluation of function predictions by pfp, esg,and psi-blast for moonlighting proteins. BMC Proc. 6, S5 (2012). 23173871 10.1186/1753-6561-6-S7-S5 PMC3504920

[b12] Jeffery C. J. Proteins with neomorphic moonlighting functions in disease. IUBMB Life 63, 489–494 (2011). 21698752 10.1002/iub.504

[b13] Becker E., Robisson B., Chapple C. E., Guénoche A. & Brun C. Multifunctional proteins revealed by overlapping clustering in protein interaction network. Bioinformatics 28, 84–90 (2012). 22080466 10.1093/bioinformatics/btr621 PMC3244771

[b14] Ashburner M. . Gene ontology: tool for the unification of biology. the gene ontology consortium. Nature Genet. 25, 25–29 (2000). 10802651 10.1038/75556 PMC3037419

[b15] Talavera D., Robertson D. L. & Lovell S. C. Alternative splicing and protein interaction data sets. Nature Biotechnol. 31, 292–293 (2013). 23563420 10.1038/nbt.2540

[b16] Ward J. J., McGuffin L. J., Bryson K., Buxton B. F. & Jones D. T. The DISOPRED server for the prediction of protein disorder. Bioinformatics 20, 2138–2139 (2004). 15044227 10.1093/bioinformatics/bth195

[b17] Oates M. . D2P2: database of disordered protein predictions. Nucleic Acids Res. 41, D508–D516 (2013). 23203878 10.1093/nar/gks1226 PMC3531159

[b18] Van Roey K. . Short linear motifs: ubiquitous and functionally diverse protein interaction modules directing cell regulation. Chem. Rev. 114, 6733–6778 (2014). 24926813 10.1021/cr400585q

[b19] Van Roey K., Dinkel H., Weatheritt R. J., Gibson T. J. & Davey N. E. The switches.elm resource: a compendium of conditional regulatory interaction interfaces. Sci. Signal. 6, rs7 (2013). 10.1126/scisignal.2003345 23550212

[b20] McKusick-Nathans Institute of Genetic Medicine, J. H. U. Online Mendelian Inheritance in Man, omim (2013). URL http://www.omim.org Accessed on May 2013]. .

[b21] Futreal P. A. . A census of human cancer genes. Nat. Rev. Cancer 4, 177–183 (2004). 14993899 10.1038/nrc1299 PMC2665285

[b22] Rolland T. . A proteome-scale map of the human interactome network. Cell 159, 1212–1226 (2014). 25416956 10.1016/j.cell.2014.10.050 PMC4266588

[b23] Zaoui K., Benseddik K., Daou P., Salaün D. & Badache A. Erbb2 receptor controls microtubule capture by recruiting acf7 to the plasma membrane of migrating cells. Proc. Natl Acad. Sci. USA 107, 18517–18522 (2010). 20937854 10.1073/pnas.1000975107 PMC2972954

[b24] Li L.-Y. . Nuclear erbb2 enhances translation and cell growth by activating transcription of ribosomal rna genes. Cancer Res. 71, 4269–4279 (2011). 21555369 10.1158/0008-5472.CAN-10-3504 PMC3117049

[b25] Holzmann J. . Rnase p without rna: identification and functional reconstitution of the human mitochondrial trna processing enzyme. Cell 135, 462–474 (2008). 18984158 10.1016/j.cell.2008.09.013

[b26] Sudol M. & Harvey K. F. Modularity in the hippo signaling pathway. Trends Biochem. Sci. 35, 627–633 (2010). 20598891 10.1016/j.tibs.2010.05.010

[b27] Monferran S., Muller C., Mourey L., Frit P. & Salles B. The membrane-associated form of the dna repair protein ku is involved in cell adhesion to fibronectin. J. Mol. Biol. 337, 503–511 (2004). 15019772 10.1016/j.jmb.2004.01.057

[b28] Hartwell L. H., Hopfield J. J., Leibler S. & Murray A. W. From molecular to modular cell biology. Nature 402, C47–C52 (1999). 10591225 10.1038/35011540

[b29] Brun C. . Functional classification of proteins for the prediction of cellular function from a protein-protein interaction network. Genome Biol. 5, R6 (2003). 14709178 10.1186/gb-2003-5-1-r6 PMC395738

[b30] Sharan R., Ulitsky I. & Shamir R. Network-based prediction of protein function. Mol. Syst. Biol. 3, 88 (2007). 17353930 10.1038/msb4100129 PMC1847944

[b31] Gómez A. . Do protein-protein interaction databases identify moonlighting proteins? Mol. Biosyst. 7, 2379–2382 (2011). 21677976 10.1039/c1mb05180f

[b32] Tompa P., Szàsz C. & Buday L. Structural disorder throws new light on moonlighting. Trends Biochem. Sci. 30, 484–489 (2005). 16054818 10.1016/j.tibs.2005.07.008

[b33] Dunker A. K., Cortese M. S., Romero P., Iakoucheva L. M. & Uversky V. N. Flexible nets. the roles of intrinsic disorder in protein interaction networks. FEBS J. 272, 5129–5148 (2005). 16218947 10.1111/j.1742-4658.2005.04948.x

[b34] Patil A., Kinoshita K. & Nakamura H. Domain distribution and intrinsic disorder in hubs in the human protein-protein interaction network. Protein Sci. 19, 1461–1468 (2010). 20509167 10.1002/pro.425 PMC2923499

[b35] Hernández S. . Do moonlighting proteins belong to the intrinsically disordered protein class? Proteomics Bioinformatics 5, 262–264 (2012).

[b36] Davey N. E. . Attributes of short linear motifs. Mol. Biosyst. 8, 268–281 (2012). 21909575 10.1039/c1mb05231d

[b37] Fuxreiter M., Tompa P. & Simon I. Local structural disorder imparts plasticity on linear motifs. Bioinformatics 23, 950–956 (2007). 17387114 10.1093/bioinformatics/btm035

[b38] Aranda B. . Psicquic and psiscore: accessing and scoring molecular interactions. Nat. Methods 8, 528–529 (2011). 21716279 10.1038/nmeth.1637 PMC3246345

[b39] Prieto C. & Rivas J. D. L. Apid: Agile protein interaction dataanalyzer. Nucleic Acids Res. 34, W298–W302 (2006). 16845013 10.1093/nar/gkl128 PMC1538863

[b40] Chatr-Aryamontri A. . The biogrid interaction database: 2013 update. Nucleic Acids Res. 41, D816–D823 (2013). 23203989 10.1093/nar/gks1158 PMC3531226

[b41] Kerrien S. . The intact molecular interaction database in 2012. Nucleic Acids Res. 40, D841–D846 (2012). 22121220 10.1093/nar/gkr1088 PMC3245075

[b42] Salwinski L. . The database of interacting proteins: 2004 update. Nucleic Acids Res. 32, D449–D451 (2004). 14681454 10.1093/nar/gkh086 PMC308820

[b43] Ceol A. . Mint, the molecular interaction database: 2009 update. Nucleic Acids Res. 38, D532–D539 (2010). 19897547 10.1093/nar/gkp983 PMC2808973

[b44] Chautard E., Ballut L., Thierry-Mieg N. & Ricard-Blum S. Matrixdb, a database focused on extracellular protein-protein and protein-carbohydrate interactions. Bioinformatics 25, 690–691 (2009). 19147664 10.1093/bioinformatics/btp025 PMC2647840

[b45] Croft D. . Reactome: a database of reactions, pathways and biological processes. Nucleic Acids Res. 39, D691–D697 (2011). 21067998 10.1093/nar/gkq1018 PMC3013646

[b46] Lynn D. J. . Innatedb: facilitating systems-level analyses of the mammalian innate immune response. Mol. Syst. Biol. 4, 218 (2008). 18766178 10.1038/msb.2008.55 PMC2564732

[b47] Elkon R. . Spike-a database, visualization and analysis tool of cellular signaling pathways. BMC Bioinformatics 9, 110 (2008). 18289391 10.1186/1471-2105-9-110 PMC2263022

[b48] Lange P. F. & Overall C. M. Topfind, a knowledgebase linking protein termini with function. Nat. Methods 8, 703–704 (2011). 21822272 10.1038/nmeth.1669

[b49] Fu L., Niu B., Zhu Z., Wu S. & Li W. Cd-hit: accelerated for clustering the next-generation sequencing data. Bioinformatics 28, 3150–3152 (2012). 23060610 10.1093/bioinformatics/bts565 PMC3516142

[b50] Skunca N., Altenhoff A. & Dessimoz C. Quality of computationally inferred gene ontology annotations. PLoS Comp. 8, e1002533 (2012). 10.1371/journal.pcbi.1002533 PMC3364937 22693439

[b51] Csardi G. & Nepusz T. The igraph software package for complex network research. InterJournal, Complex Systems, 1695 (2006).

[b52] Mistry J., Bateman A. & Finn R. D. Predicting active site residue annotations in the pfam database. BMC Bioinformatics 8, 298–312 (2007). 17688688 10.1186/1471-2105-8-298 PMC2025603

[b53] Su A. I. . A gene atlas of the mouse and human protein-encoding transcriptomes. Proc. Natl Acad. Sci. USA 101, 6062–6067 (2004). 15075390 10.1073/pnas.0400782101 PMC395923

[b54] Xue Y. . Gps 2.0, a tool to predict kinase-specific phosphorylation sites in hierarchy. Mol. Cell Proteomics 7, 1598–1608 (2008). 18463090 10.1074/mcp.M700574-MCP200 PMC2528073

[b55] Notredame C., Higgins D. G. & Heringa J. T-coffee: a novel method for fast and accurate multiple sequence alignment. J. Mol. Biol. 302, 205–217 (2000). 10964570 10.1006/jmbi.2000.4042

[b56] Flicek P. . Ensembl 2014. Nucleic Acids Res. 42, D749–D755 (2014). 24316576 10.1093/nar/gkt1196 PMC3964975

[b57] Puntervoll P. . Elm server: a new resource for investigating short functional sites in modular eukaryotic proteins. Nucleic Acids Res. 31, 3625–3630 (2003). 12824381 10.1093/nar/gkg545 PMC168952

