# Supplementary material for: Extreme multifunctional proteins identified from a human protein interaction network
Source: Nat Commun. 2015 Jun 9;6:7412. doi: 10.1038/ncomms8412 (PMC4468855; doi:10.1038/ncomms8412)
Supplement: Supplementary Information — Supplementary Figures 1-32 and Supplementary Note 1 [file ncomms8412-s1.pdf]

Contents

1 Analyses of the full interactome **2**

1.1 Supplementary Figure 1: Clusters . . . 3
1.2 Supplementary Figure 2: Betweeness . . . 4
1.3 Supplementary Figure 3: Shortest Paths . . . 5
1.4 Supplementary Figure 4: Length . . . 6
1.5 Supplementary Figure 5: Conservation . . . 7
1.6 Supplementary Figure 6: Stretches of consecutive disordered residues of different lengths . . . 8
1.7 Protein disorder as predicted by different software . . . 9
1.7.1 Supplementary Figure 7: Espritz-D . . . 10
1.7.2 Supplementary Figure 8: Espritz-N . . . 11
1.7.3 Supplementary Figure 9: Espritz-X . . . 12
1.7.4 Supplementary Figure 10: IUPred-A . . . 13
1.7.5 Supplementary Figure 11: IUPred-L . . . 14
1.7.6 Supplementary Figure 12: IUPred-S . . . 15
1.7.7 Supplementary Figure 13: PrDOS . . . 16
1.7.8 Supplementary Figure 14: PV2 . . . 17
1.7.9 Supplementary Figure 15: VLXT . . . 18
1.7.10 Supplementary Figure 16: VSL2b . . . 19
1.8 Supplementary Figure 17: ELMs . . . 20
1.9 Supplementary Figure 18: Annotations . . . 21
1.10 Supplementary Figure 19: Expression . . . 22

2 Results on unbiased network **23**

2.1 Supplementary Figure 20: Annotations . . . 24
2.2 Supplementary Figure 21: Betweeness . . . 25
2.3 Supplementary Figure 22: Clusters . . . 26
2.4 Supplementary Figure 23: Conservation . . . 27
2.5 Supplementary Figure 24: Degree . . . 28
2.6 Supplementary Figure 25: Domains . . . 29
2.7 Supplementary Figure 26: Disorder . . . 30
2.8 Supplementary Figure 27: Expression . . . 31
2.9 Supplementary Figure 28: Isoforms . . . 32
2.10 Supplementary Figure 29: Length . . . 33
2.11 Supplementary Figure 30: Phosphorylation . . . 34
2.12 Supplementary Figure 31: Shortest Paths . . . 35

3 Supplementary Note **36**

1 Analyses of the full interactome

Each of the figures below shows our candidates compared to all nodes (Network), network hubs (nodes whose degree is at least twice the network average), nodes that belong to multiple clusters but are not candidates (Multi NC), all nodes that belong to multiple clusters (Multi), nodes that belong to one cluster alone (Mono) and all non-candidate nodes (NC). The width of the boxes is proportional to the number of nodes in each group. The numbers in parentheses under each group are the Wilcoxon p-values. Red dots indicate the mean values and yellow dots the values of known moonlighting proteins. Outliers are not shown.

1.1 Supplementary Figure 1: Clusters

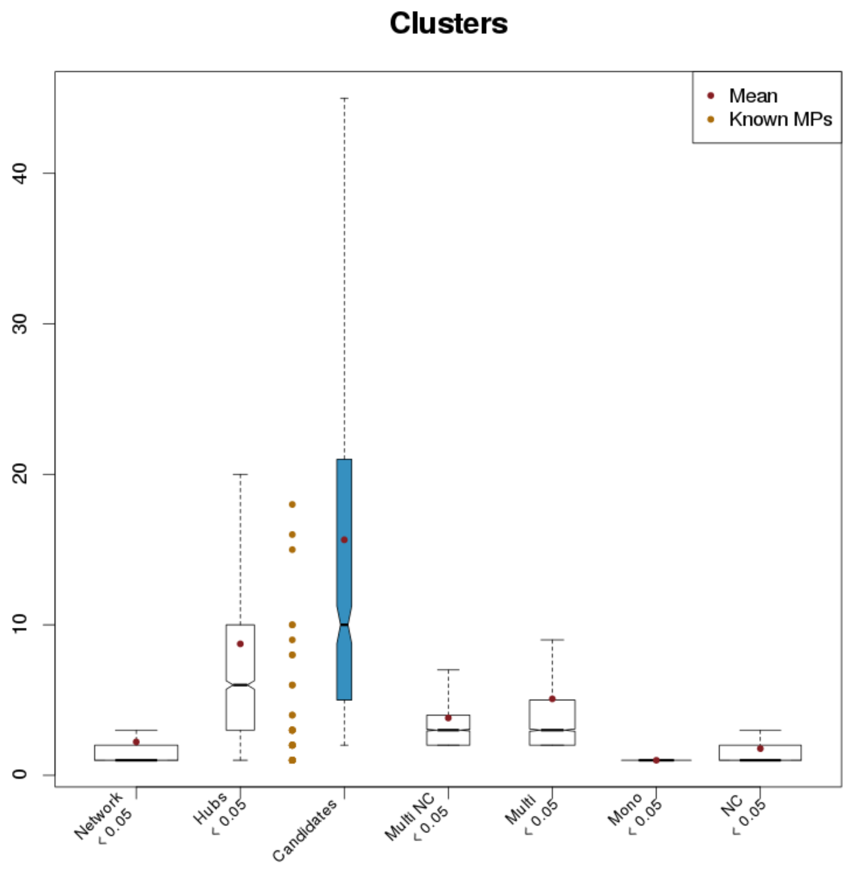

Supplementary Figure 1: The number of OCG clusters each group belongs to.

1.2 Supplementary Figure 2: Betweeness

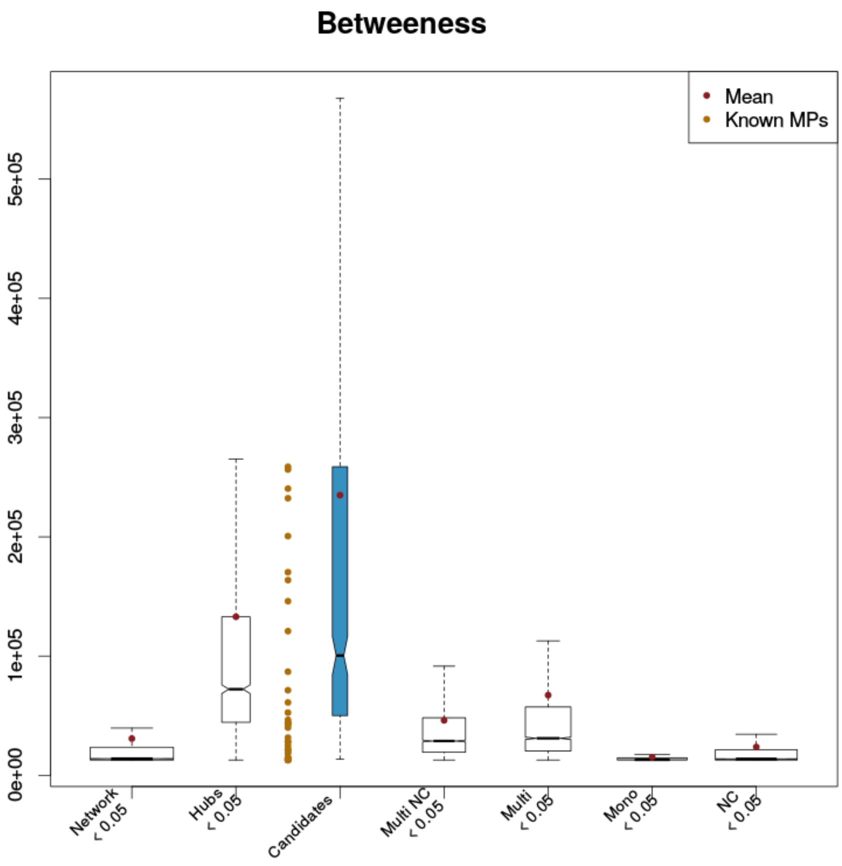

Supplementary Figure 2: Node Betweeness values.

1.3 Supplementary Figure 3: Shortest Paths

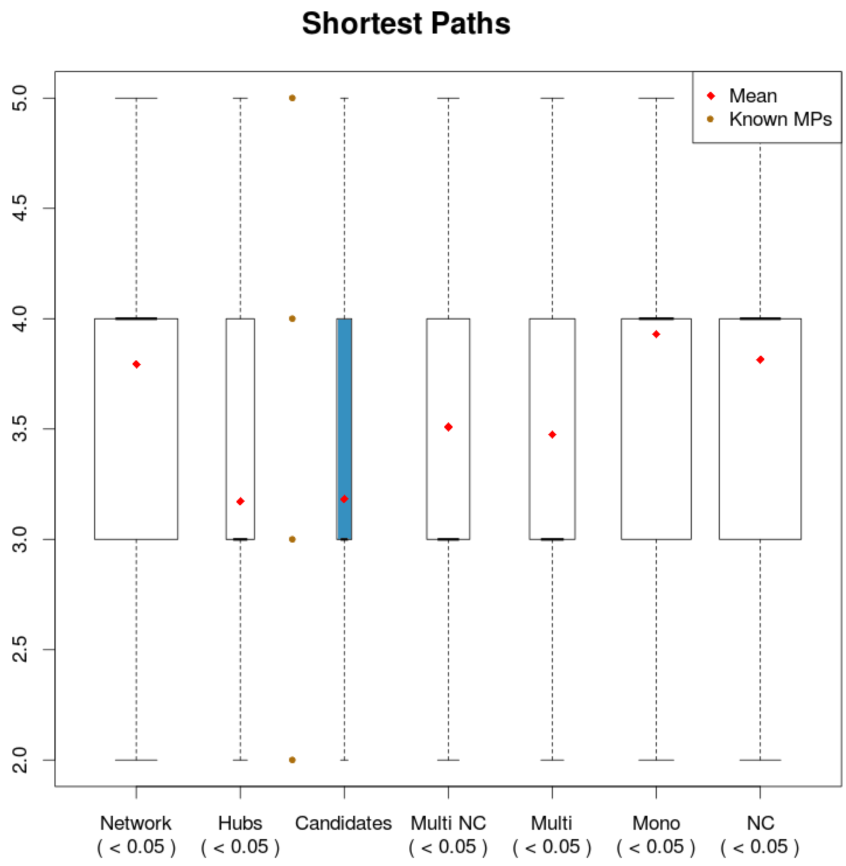

Supplementary Figure 3: Shortest Paths.

1.4 Supplementary Figure 4: Length

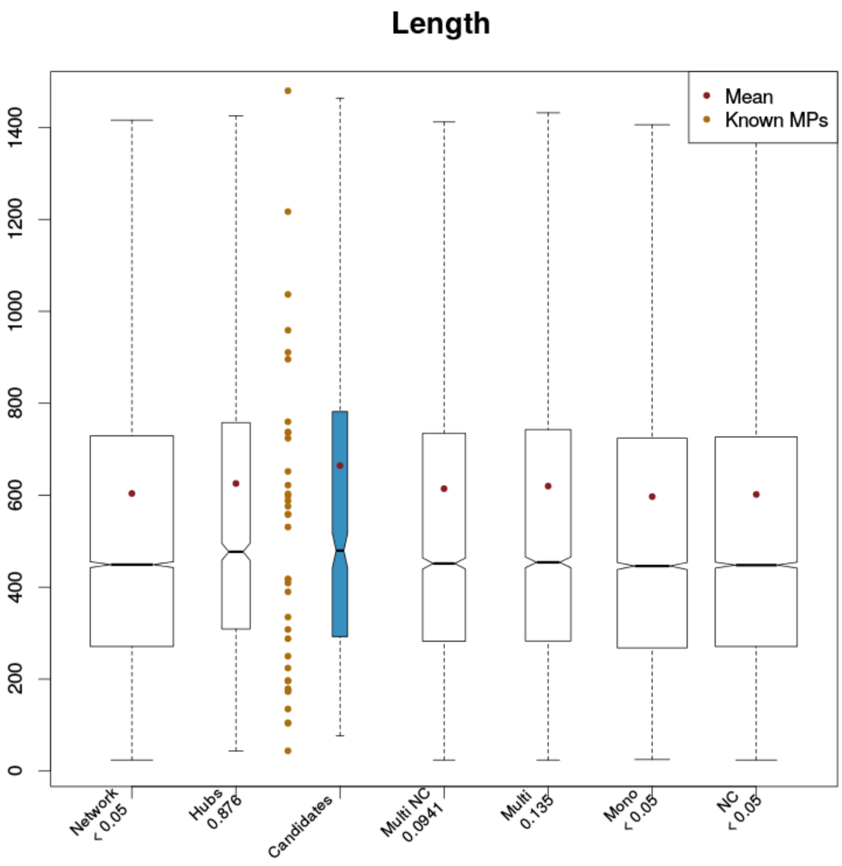

Supplementary Figure 4: Protein length.

1.5 Supplementary Figure 5: Conservation

The protein sequences of all network proteins and their annotated homologs in yeast, mouse, fly and worm were aligned against each other using t_coffee. The homologous sequences were taken from EnsEMBL. To obtain an indicative conservation value, the bit score of the alignments was divided against the length of the human homolog and the resulting values were compared.

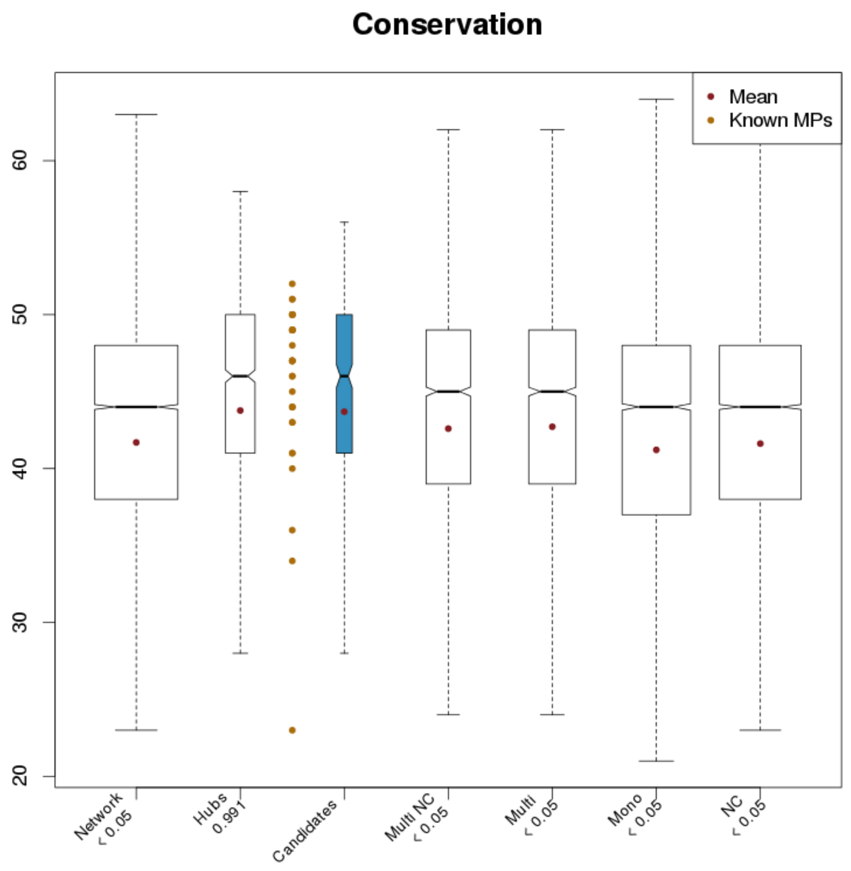

Supplementary Figure 5: Protein conservation.

1.6 Supplementary Figure 6: Stretches of consecutive disordered residues of different lengths

The number of consecutive stretches of disordered aminoacids as predicted by disopred was measured using sliding windows of different lengths. The results are shown in the figure below. Note that the candidates consistently have fewer stretches of any given length than hubs.

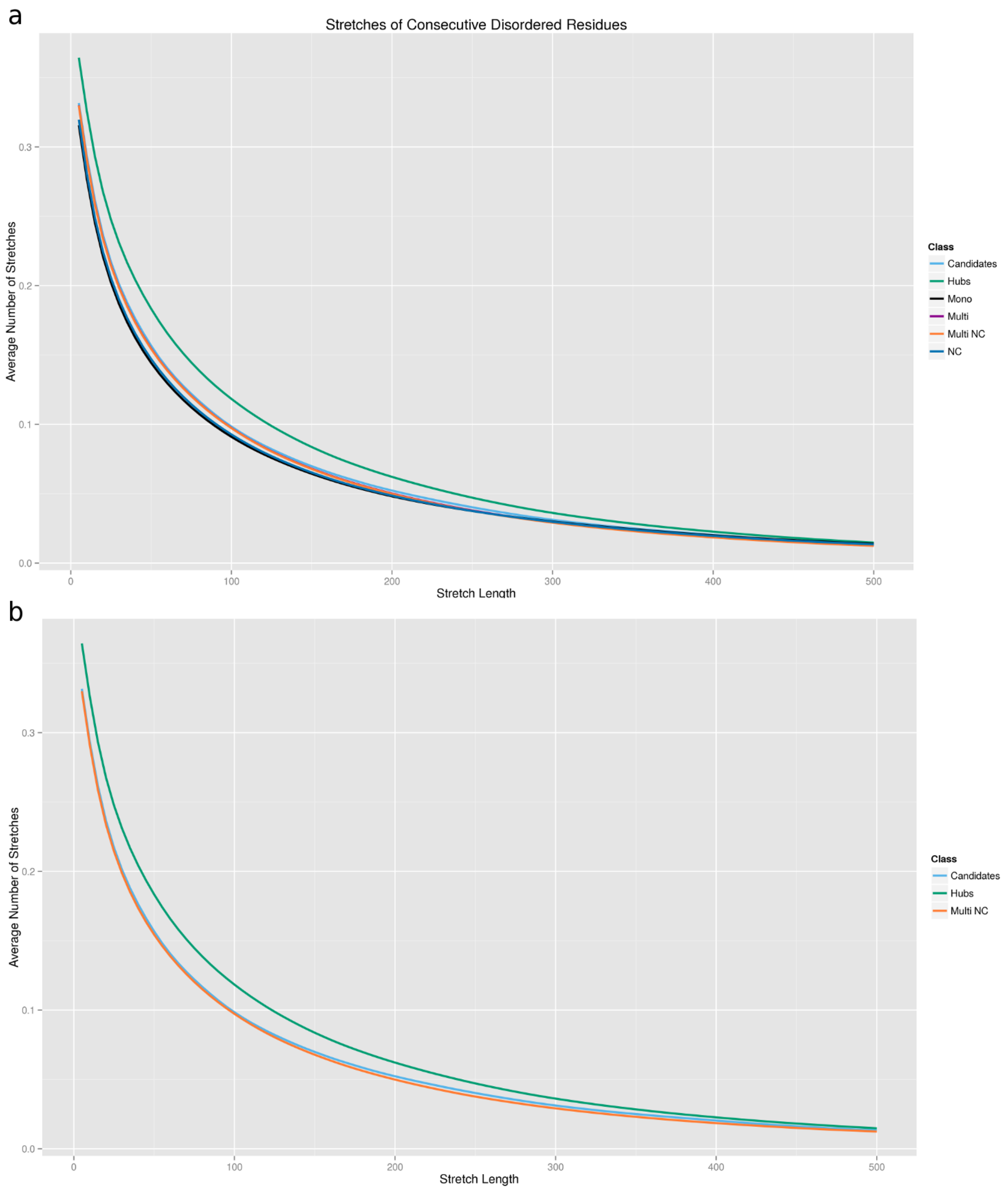

Supplementary Figure 6: Stretches of consecutive disordered residues as predicted by disopred. Figure b is figure a with only the three groups, for clarity

1.7 Protein disorder as predicted by different software

Each of the figures below shows protein disorder as calculated by different predictors for our candidates compared to all nodes (Network), network hubs (nodes whose degree is at least twice the network average), nodes that belong to multiple clusters but are not candidates (Multi NC), all nodes that belong to multiple clusters (Multi), nodes that belong to one cluster alone (Mono) and all non-candidate nodes (NC). The width of the boxes is proportional to the number of nodes in each group. The numbers in parentheses under each group are the Wilcoxon p-values. Red dots indicate the mean values and yellow dots the values of known moonlighting proteins. Outliers are not shown.

1.7.1 Supplementary Figure 7: Espritz-D

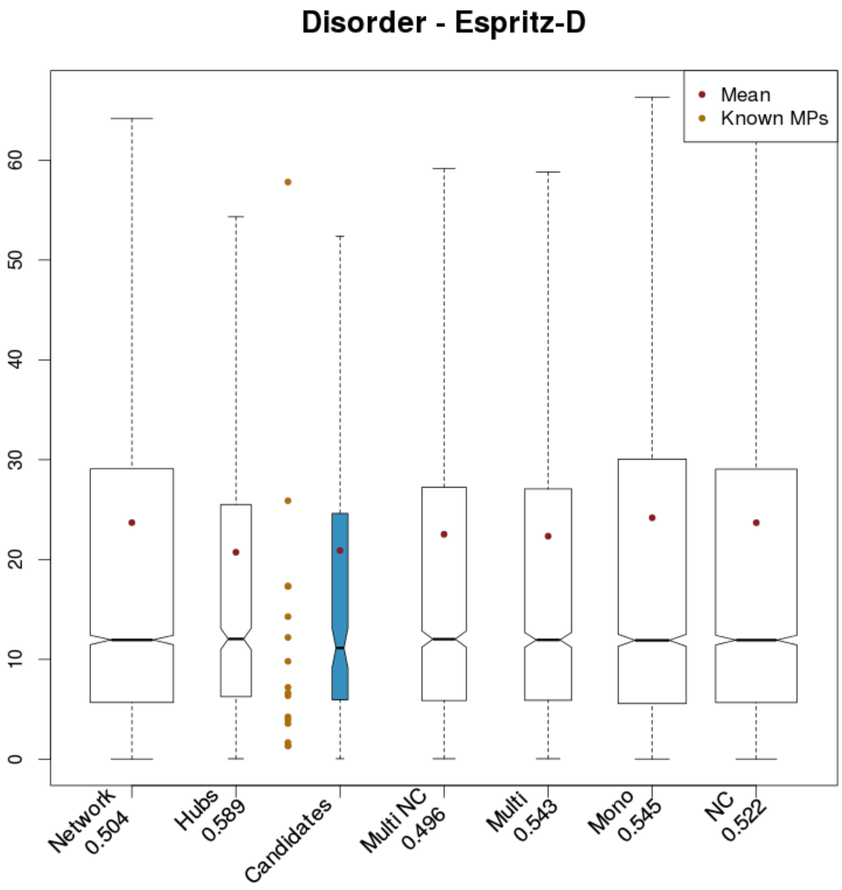

Supplementary Figure 7: Protein disorder as predicted by Espritz-D

1.7.2 Supplementary Figure 8: Espritz-N

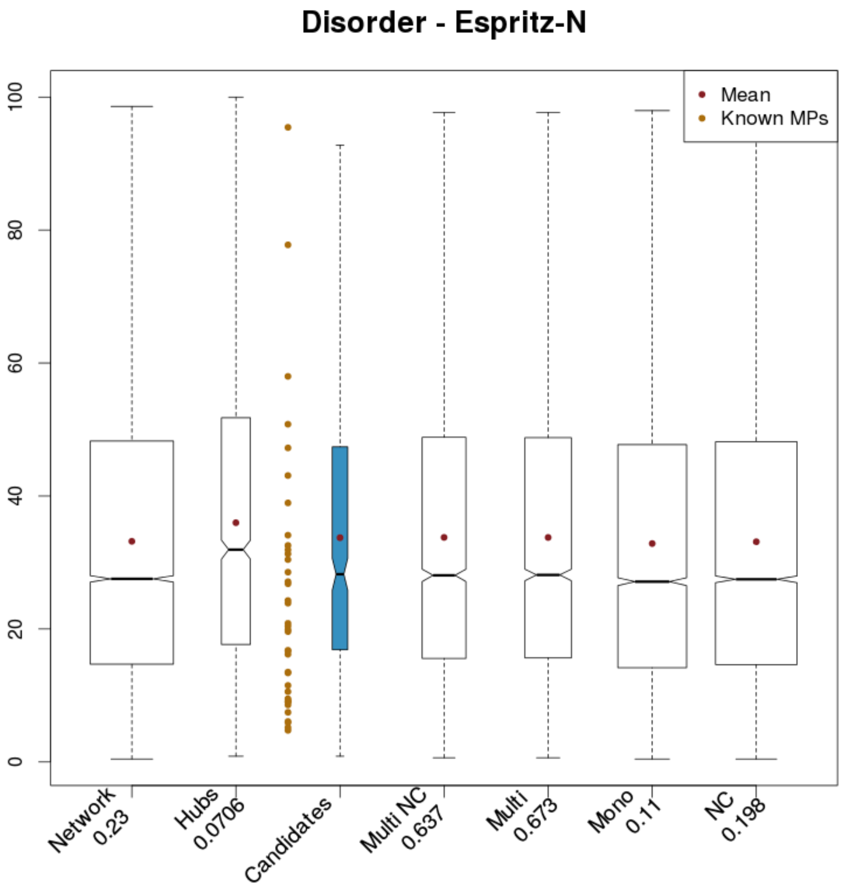

Supplementary Figure 8: Protein disorder as predicted by Espritz-N

1.7.3 Supplementary Figure 9: Espritz-X

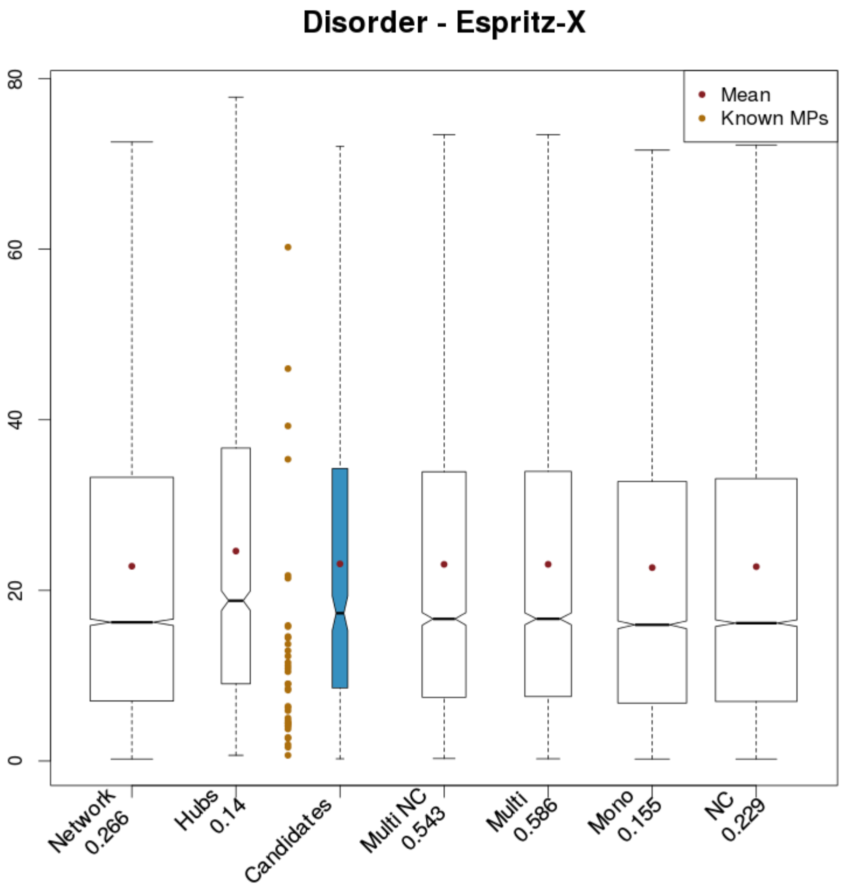

Supplementary Figure 9: Protein disorder as predicted by Espritz-X

1.7.4 Supplementary Figure 10: IUPred-A

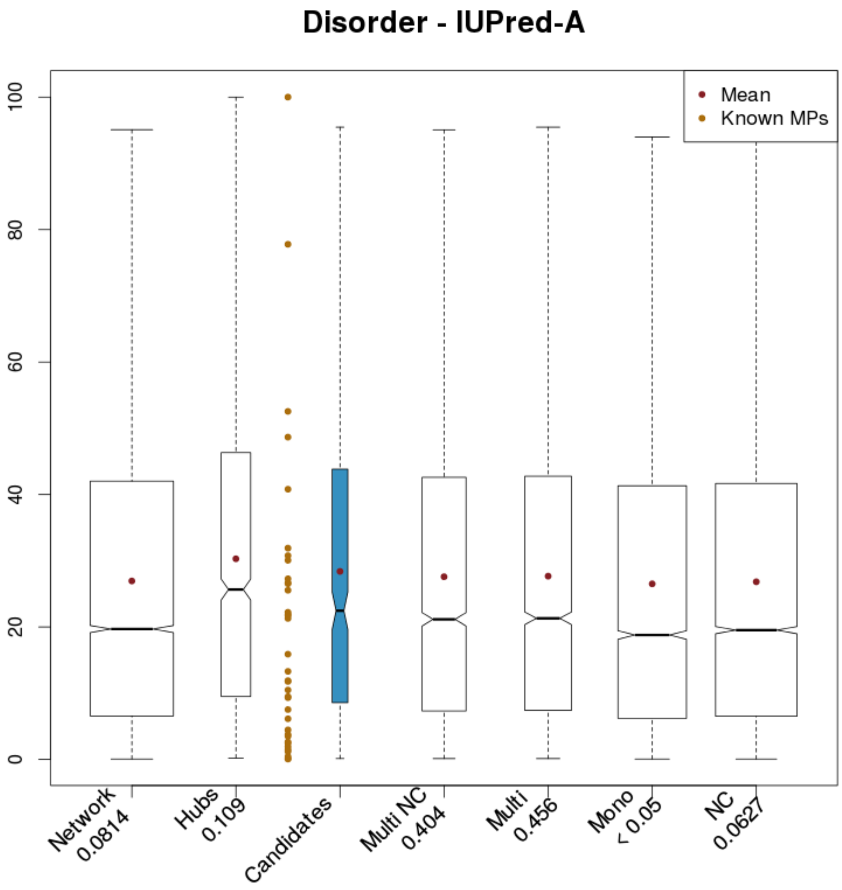

Supplementary Figure 10: Protein disorder as predicted by IUPred-A

1.7.5 Supplementary Figure 11: IUPred-L

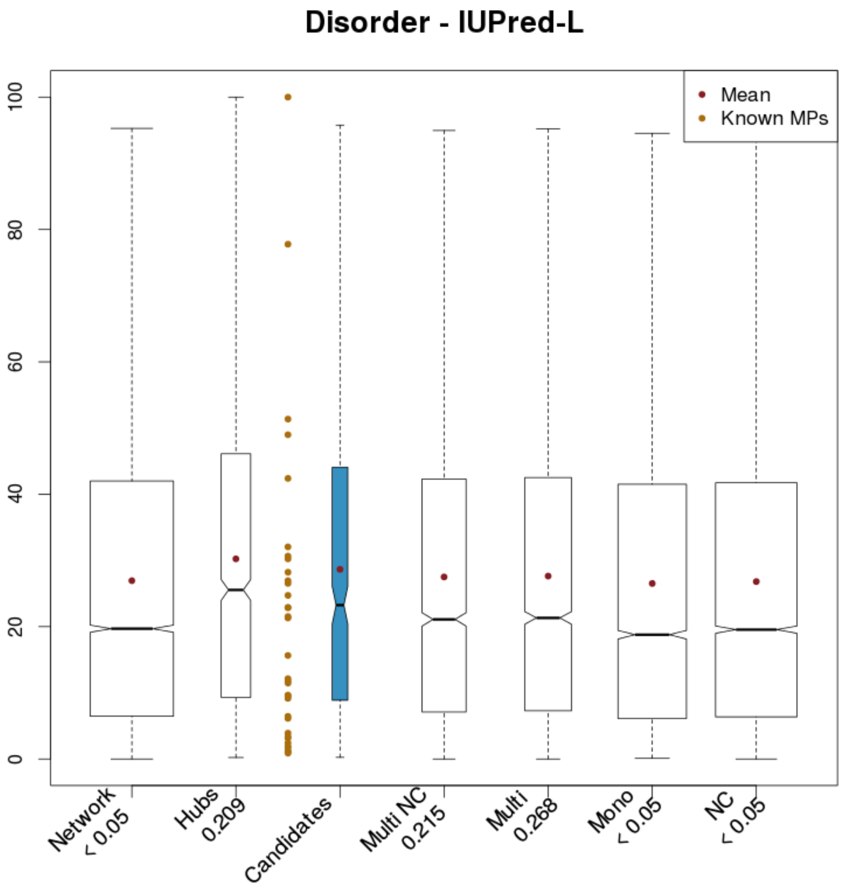

Supplementary Figure 11: Protein disorder as predicted by IUPred-L

1.7.6 Supplementary Figure 12: IUPred-S

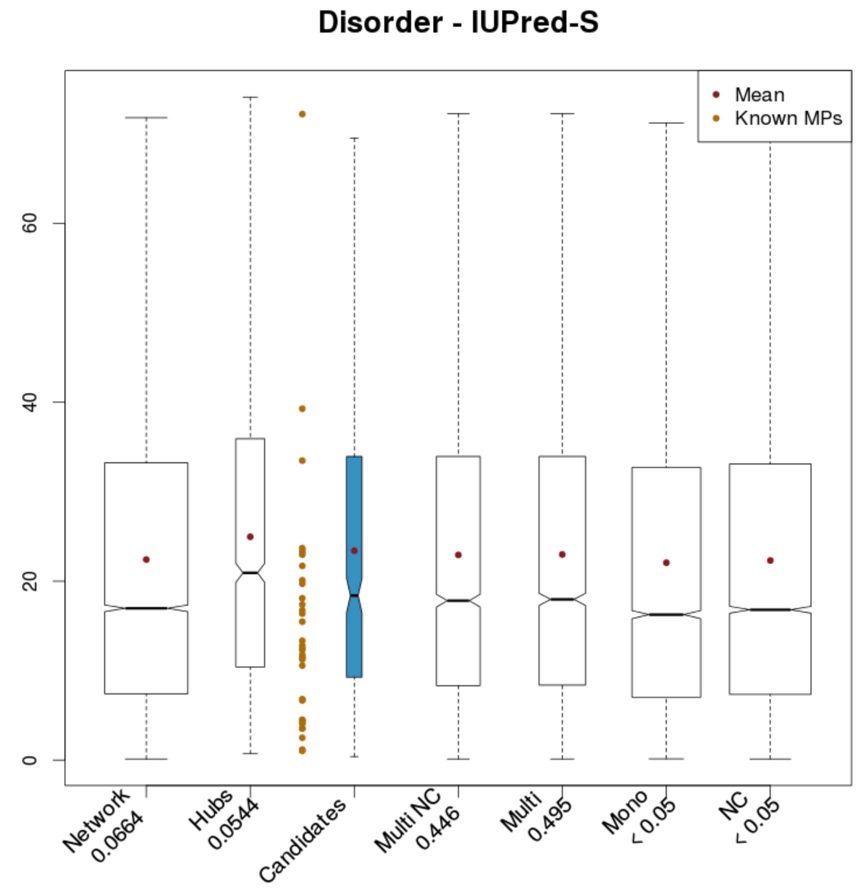

Supplementary Figure 12: Protein disorder as predicted by IUPred-S

1.7.7 Supplementary Figure 13: PrDOS

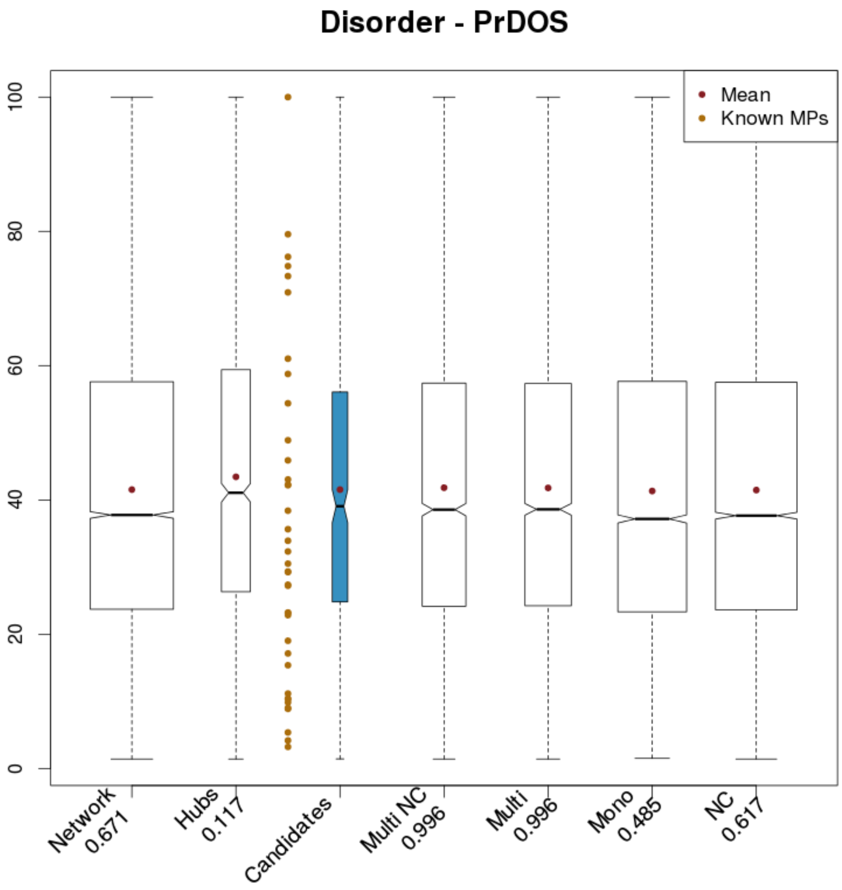

Supplementary Figure 13: Protein disorder as predicted by PrDOS

1.7.8 Supplementary Figure 14: PV2

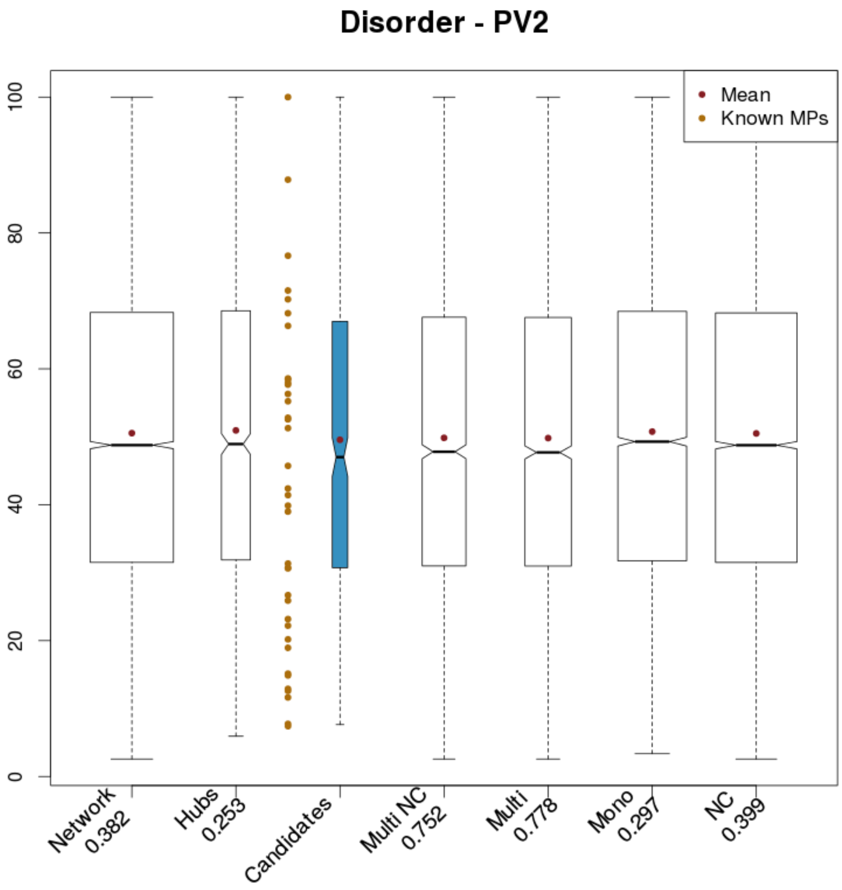

Supplementary Figure 14: Protein disorder as predicted by PV2

1.7.9 Supplementary Figure 15: VLXT

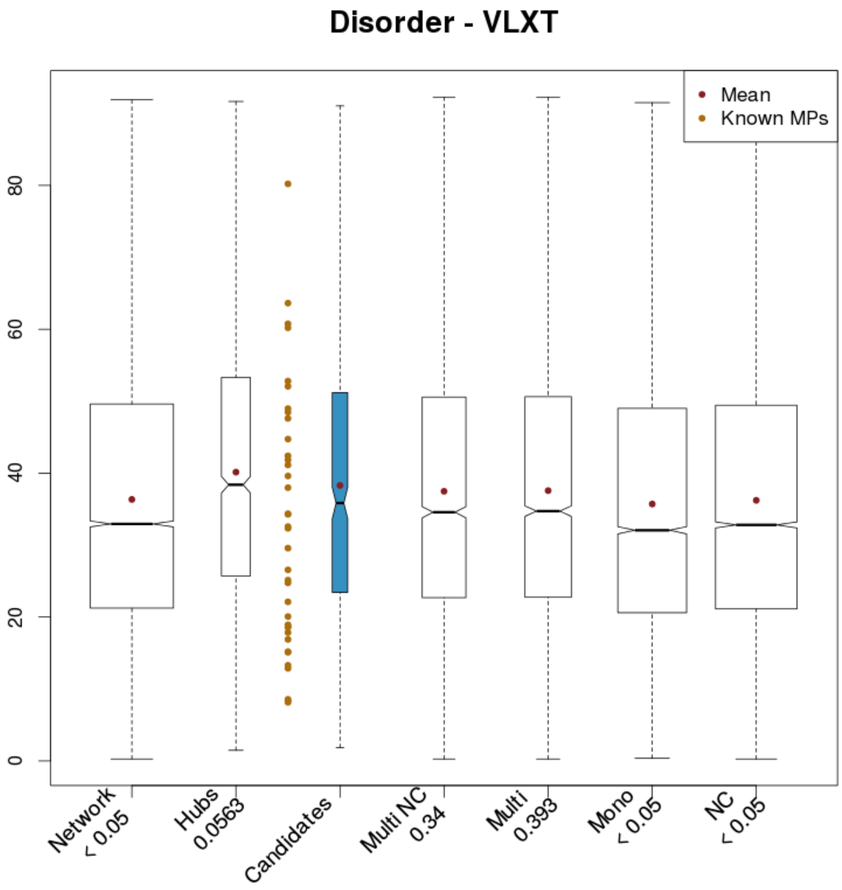

Supplementary Figure 15: Protein disorder as predicted by VLXT

1.7.10 Supplementary Figure 16: VSL2b

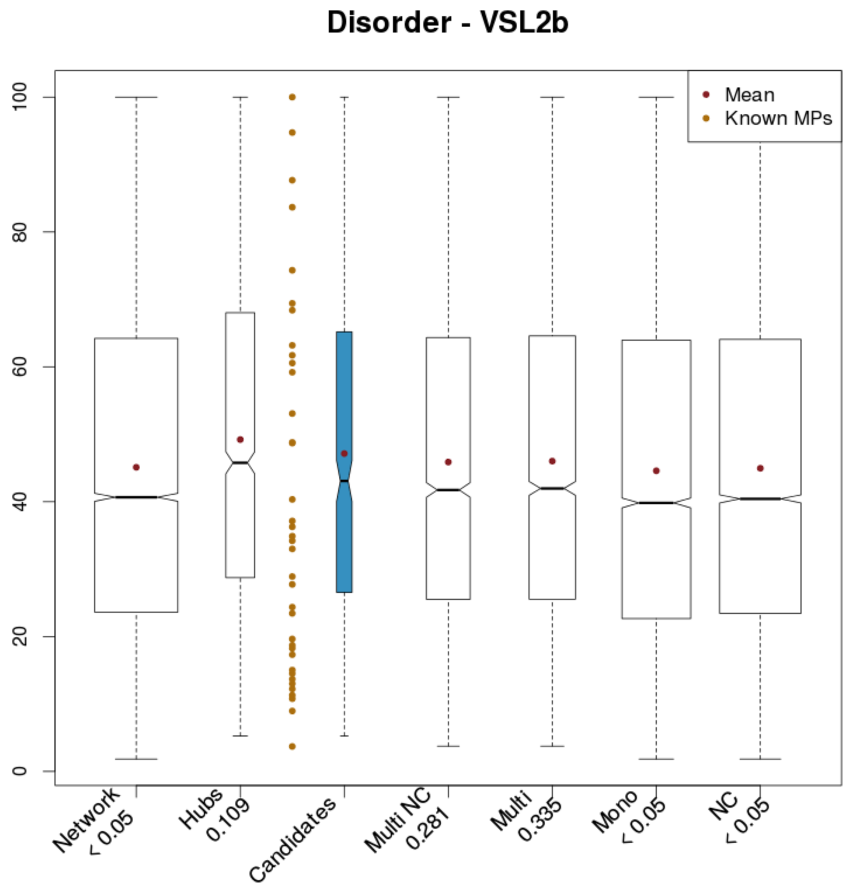

Supplementary Figure 16: Protein disorder as predicted by VSL2b

1.8 Supplementary Figure 17: ELMs

The coordinates of all known Eukaryotic Linear Motifs (ELMs) in our network's proteins were downloaded from the ELM database (http://elm.eu.org, Van Roey *et al.*, 2013). The number of ELMs that were completely within disordered regions was calculated for each protein and divided by the total number of disordered residues in that protein (to correct for differences in protein/disordered region length). The results are shown below.

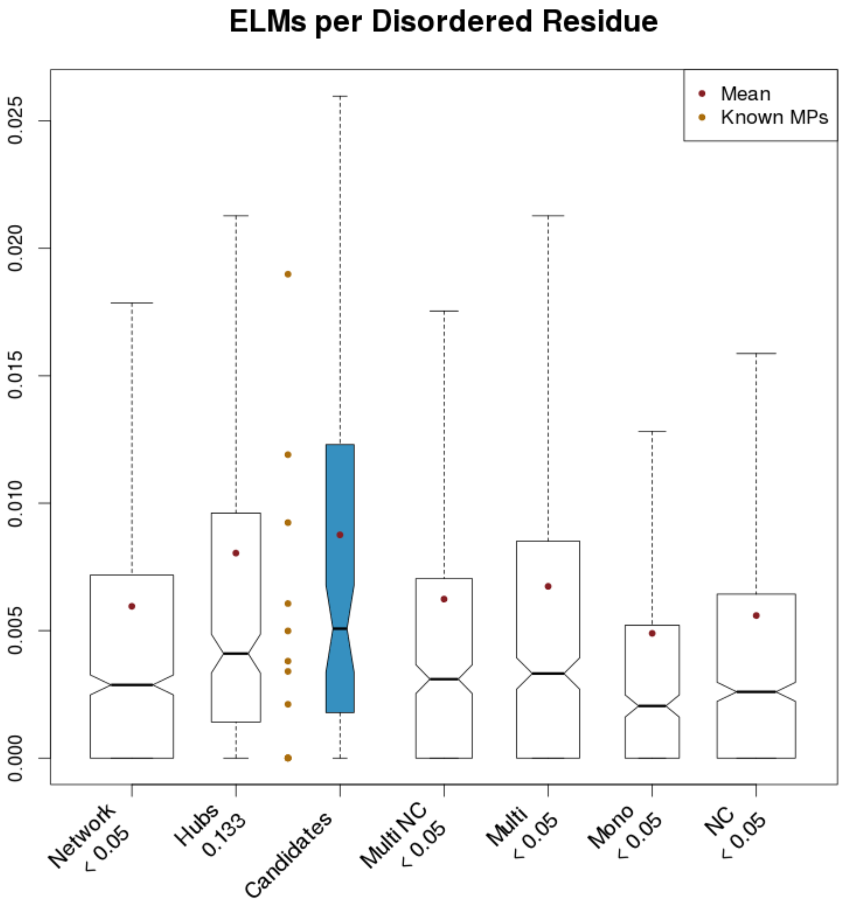

Supplementary Figure 17: The number of ELMs per disordered residue for each group.

1.9 Supplementary Figure 18: Annotations

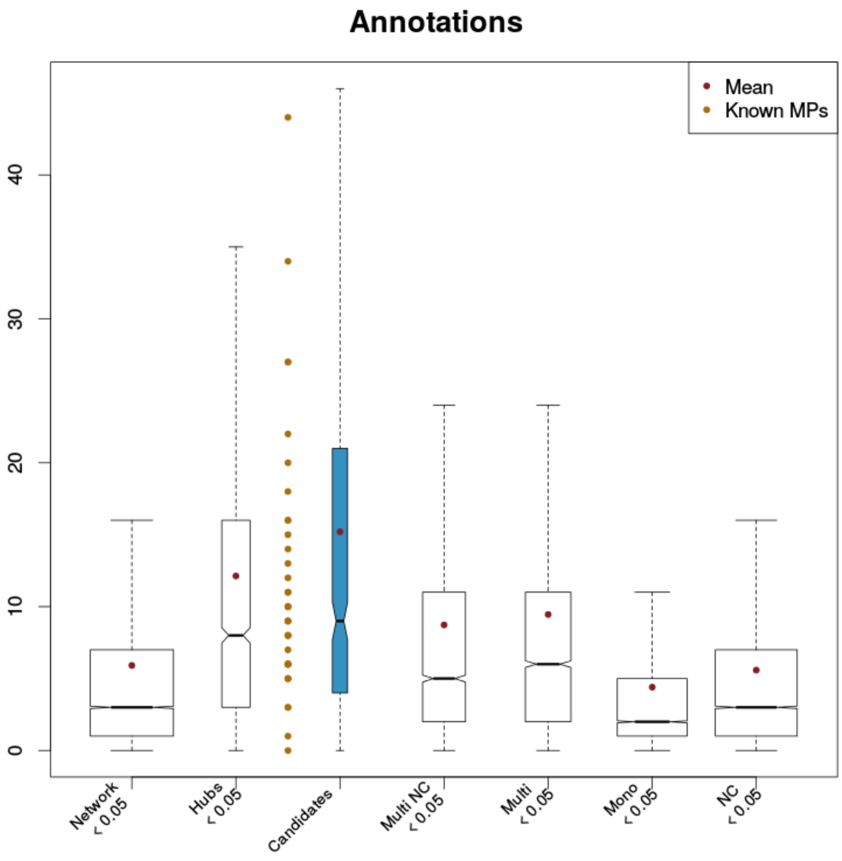

Supplementary Figure 18: Gene Ontology direct (annotations inherited from ancestor terms are not included) Biological Process annotations.

1.10 Supplementary Figure 19: Expression

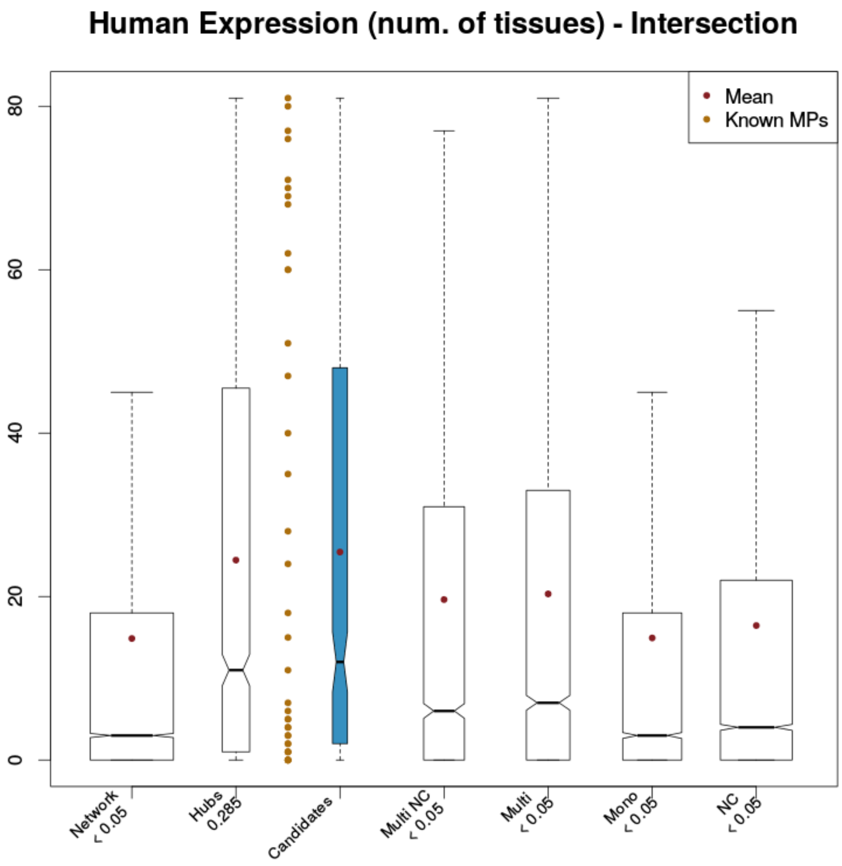

Supplementary Figure 19: The number of tissues each protein is expressed in. Expression data were taken from Su *et al.*, 2004 (see References in the main text).

2 Results on unbiased network

As a control against bias introduced by extensively studied proteins, the analyses on the human interactome were repeated on a smaller human PPI network (15617 interactions between 4494 proteins), built exclusively from large-scale yeast two-hybrid data (CCSB network; Yu, 2011). We include the results of these analyses here.

Each of the figures below shows our candidates compared to all nodes (Network), network hubs (nodes whose degree is at least twice the network average), nodes that belong to multiple clusters but are not candidates (Multi NC), all nodes that belong to multiple clusters (Multi), nodes that belong to one cluster alone (Mono) and all non-candidate nodes (NC). The width of the boxes is proportional to the number of nodes in each group. The numbers in parentheses under each group are the Wilcoxon p-values. Red dots indicate the mean values and yellow dots the values of known moonlighting proteins. Outliers are not shown.

2.1 Supplementary Figure 20: Annotations

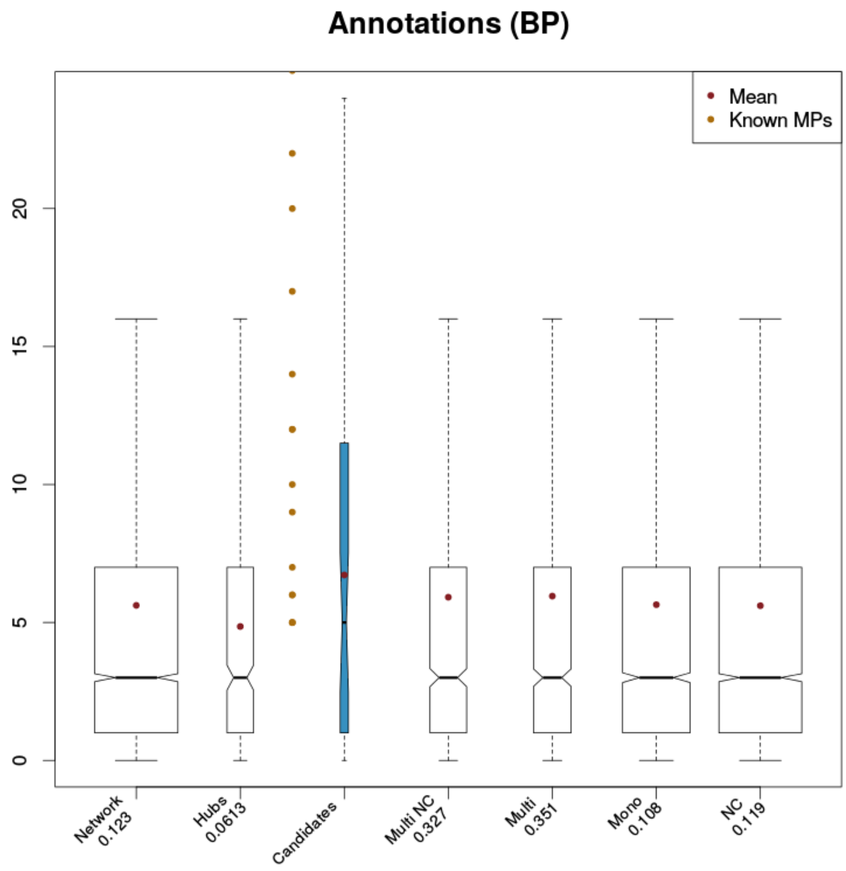

Supplementary Figure 20: Gene Ontology direct (annotations inherited from ancestor terms are not included) Biological Process annotations.

2.2 Supplementary Figure 21: Betweeness

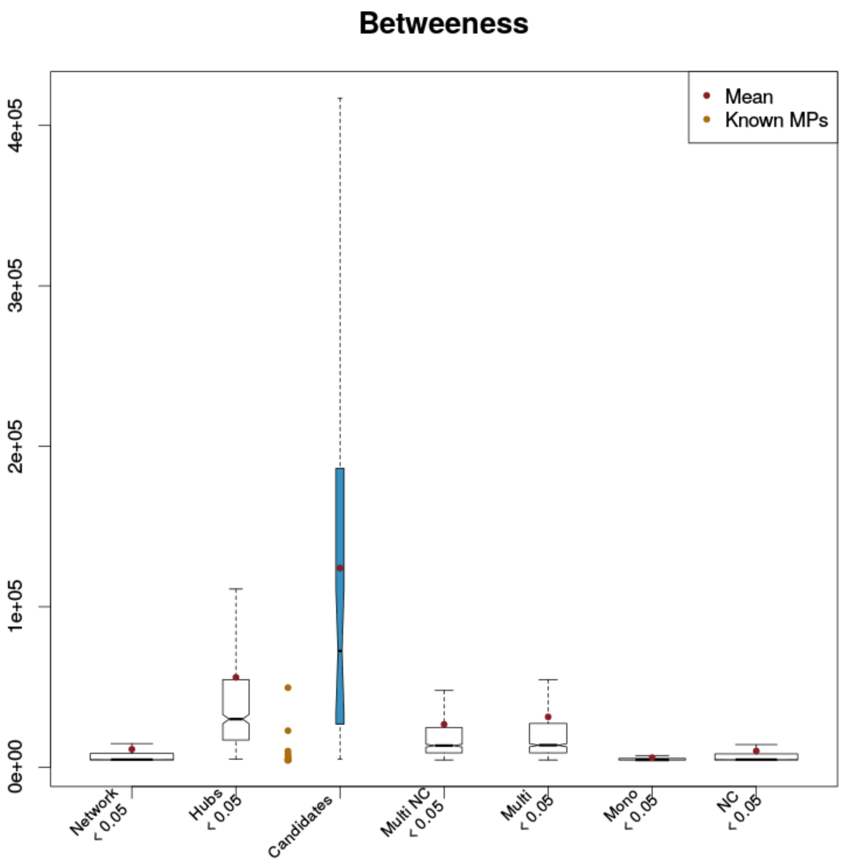

Supplementary Figure 21: Node Betweeness values.

2.3 Supplementary Figure 22: Clusters

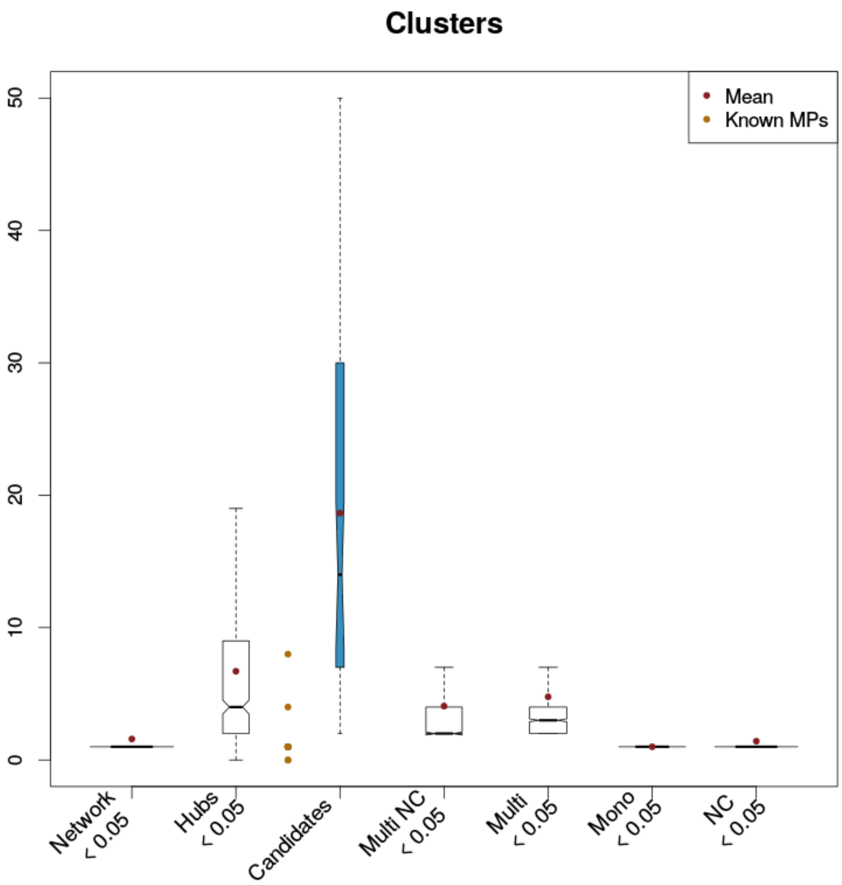

Supplementary Figure 22: The number of OCG clusters each group belongs to.

2.4 Supplementary Figure 23: Conservation

The protein sequences of all network proteins and their annotated homologs in yeast, mouse, fly and worm were aligned against each other using t_coffee. The homologous sequences were taken from EnsEMBL. To obtain an indicative conservation value, the bit score of the alignments was divided against the length of the human homolog and the resulting values were compared.

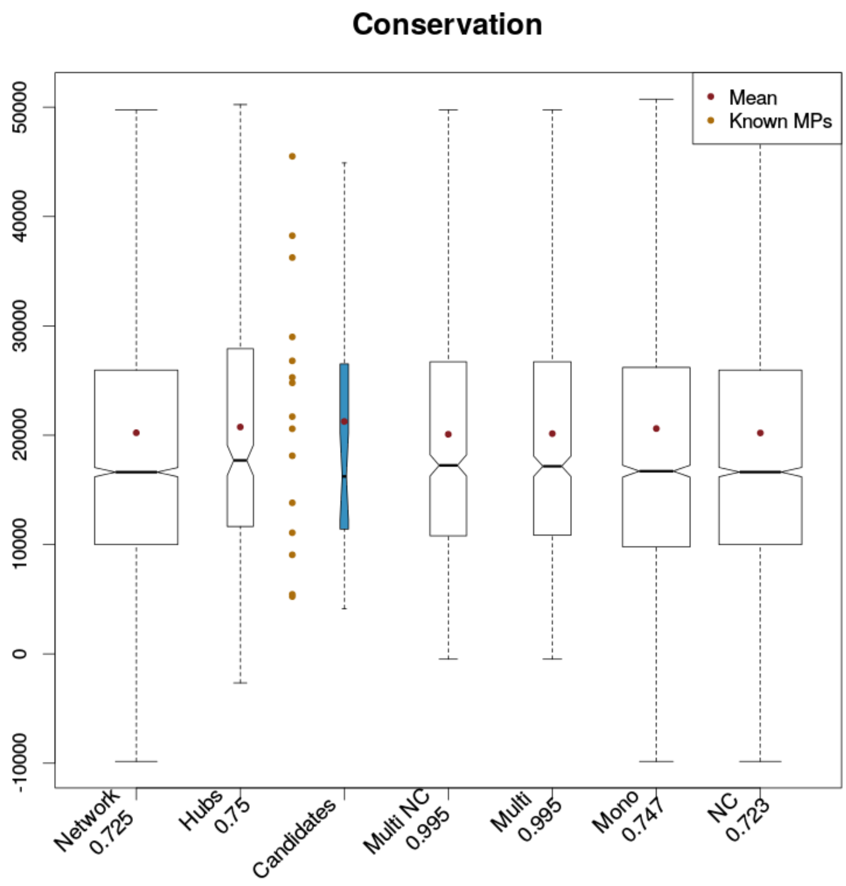

Supplementary Figure 23: Protein conservation

2.5 Supplementary Figure 24: Degree

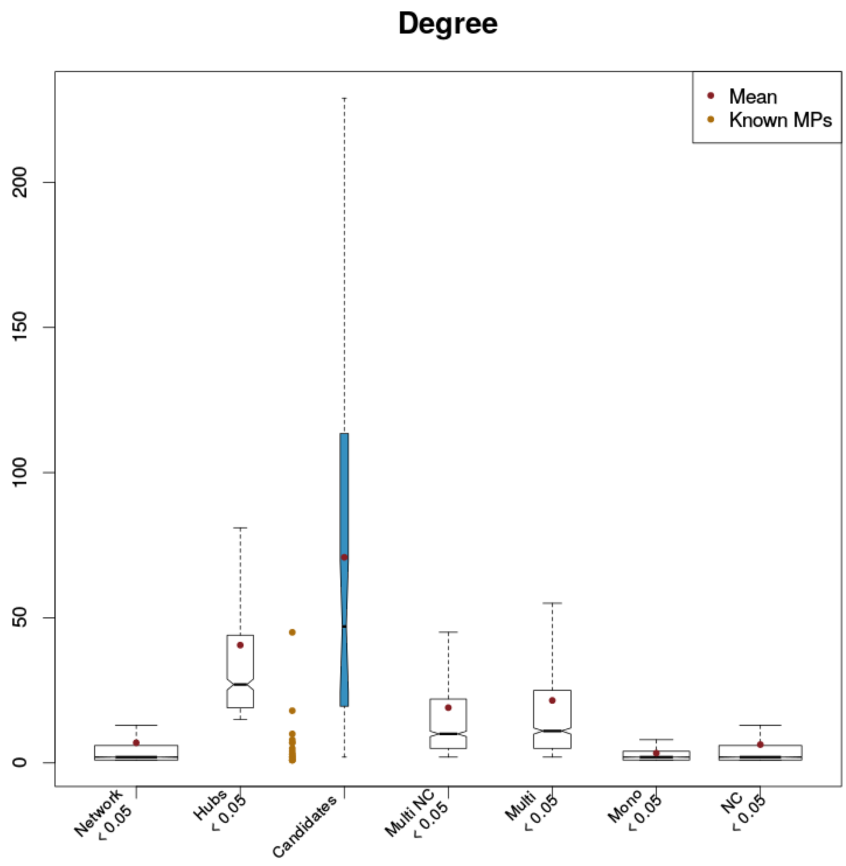

Supplementary Figure 24: Node degree.

2.6 Supplementary Figure 25: Domains

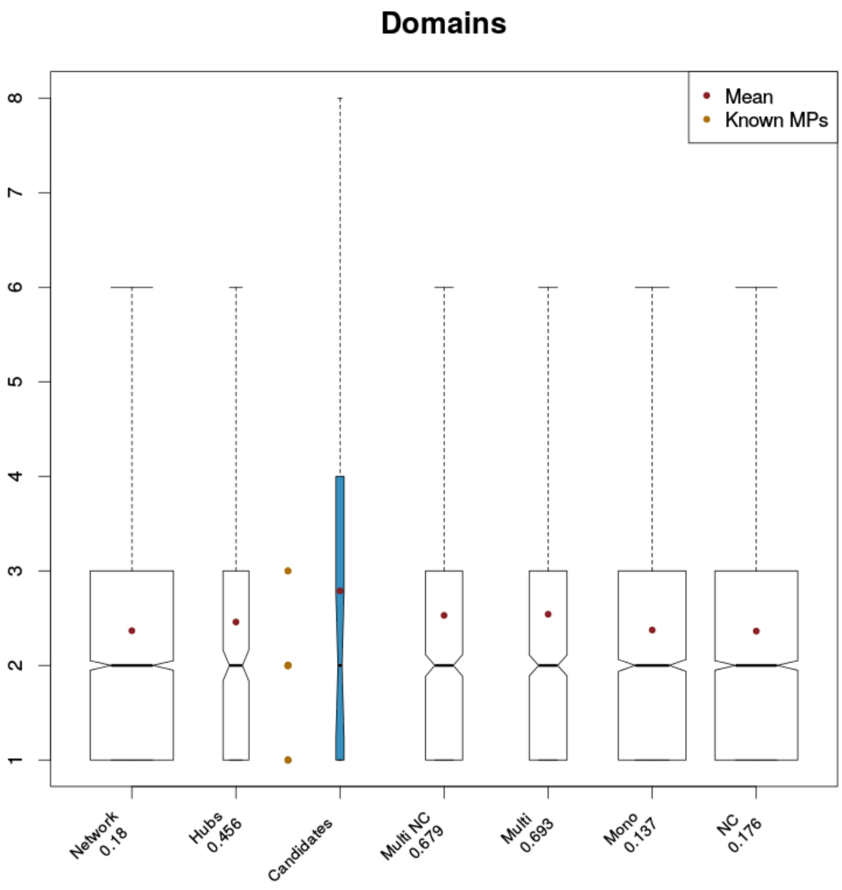

Supplementary Figure 25: The number of Pfam domains (including PfamB) predicted on each protein.

2.7 Supplementary Figure 26: Disorder

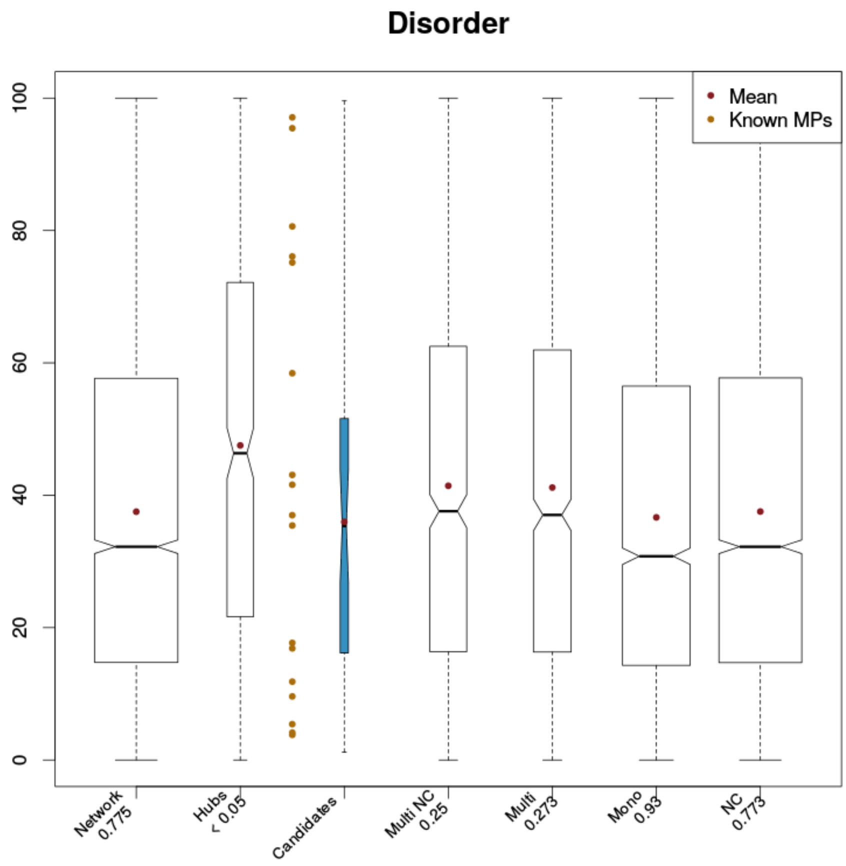

Supplementary Figure 26: Protein disorder as calculated by disopred. The numbers shown are the percentage of a protein's residues that are in disordered regions. A value of 100 means that the entire protein is intrinsically disordered.

2.8 Supplementary Figure 27: Expression

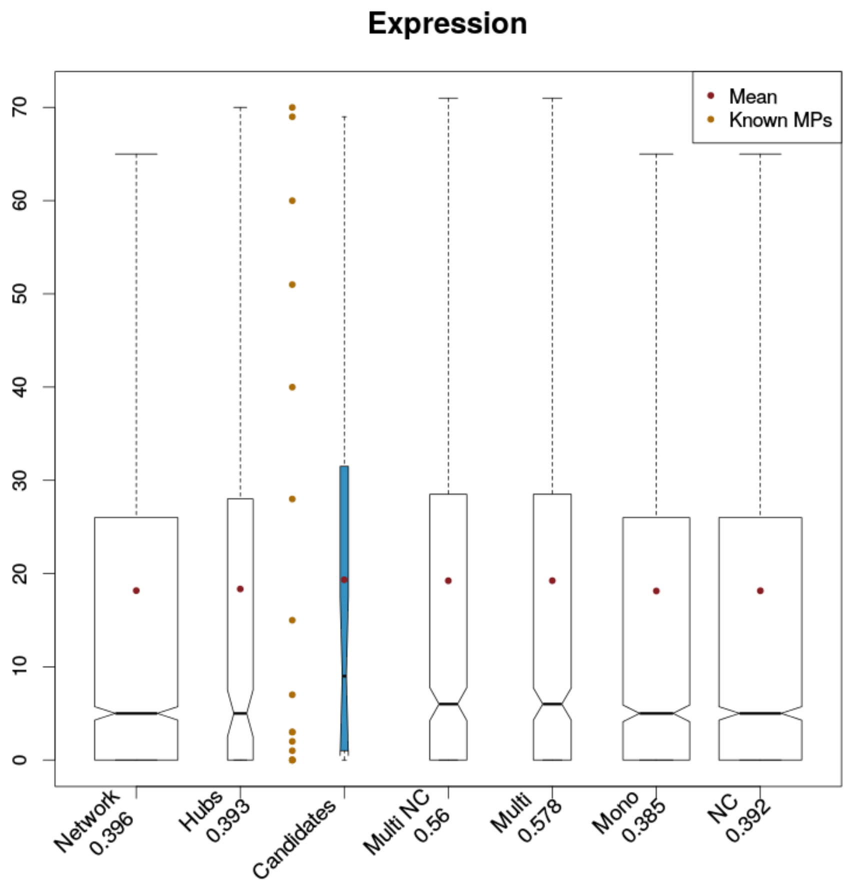

Supplementary Figure 27: The number of tissues a protein is expressed in.

2.9 Supplementary Figure 28: Isoforms

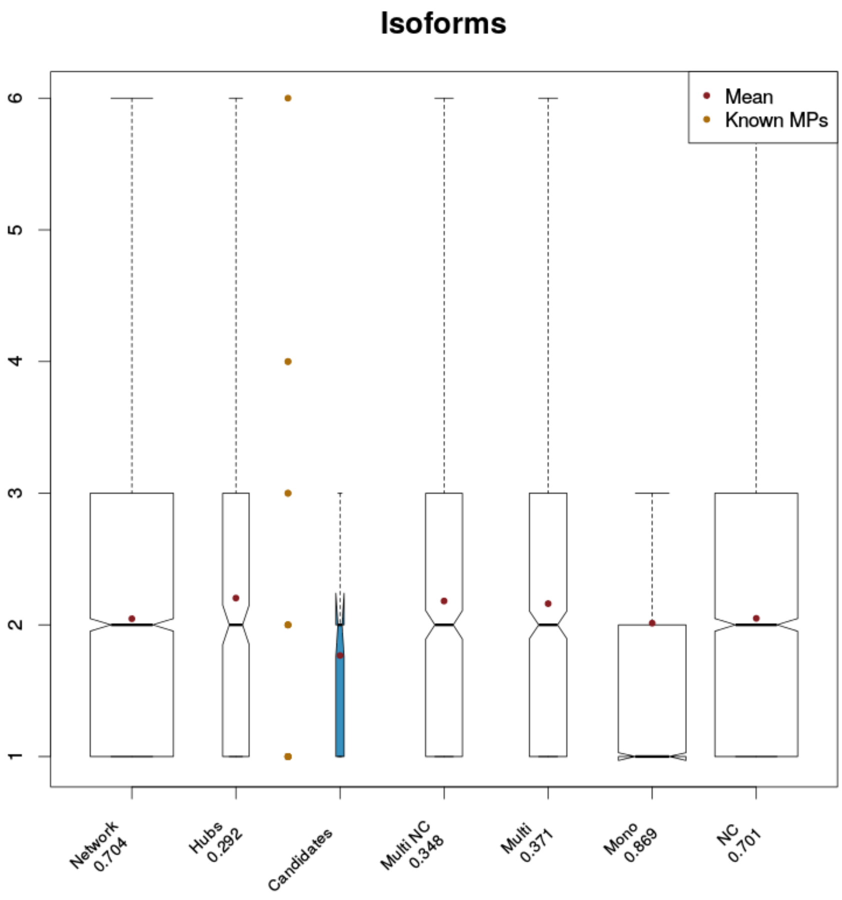

Supplementary Figure 28: Protein isoforms.

2.10 Supplementary Figure 29: Length

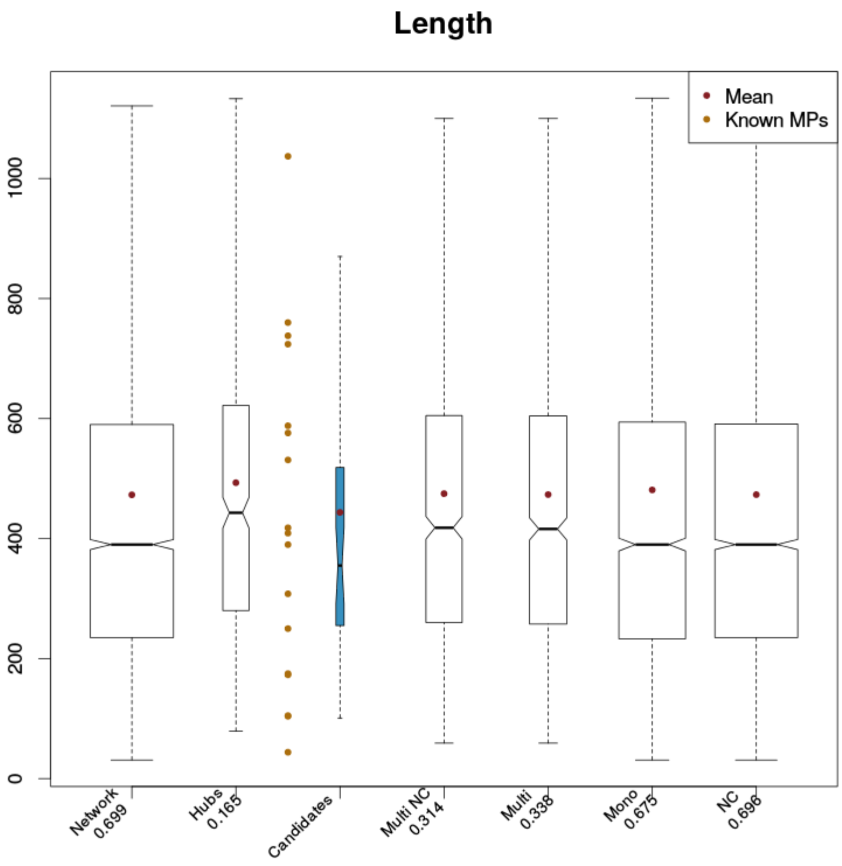

Supplementary Figure 29: Protein length.

2.11 Supplementary Figure 30: Phosphorylation

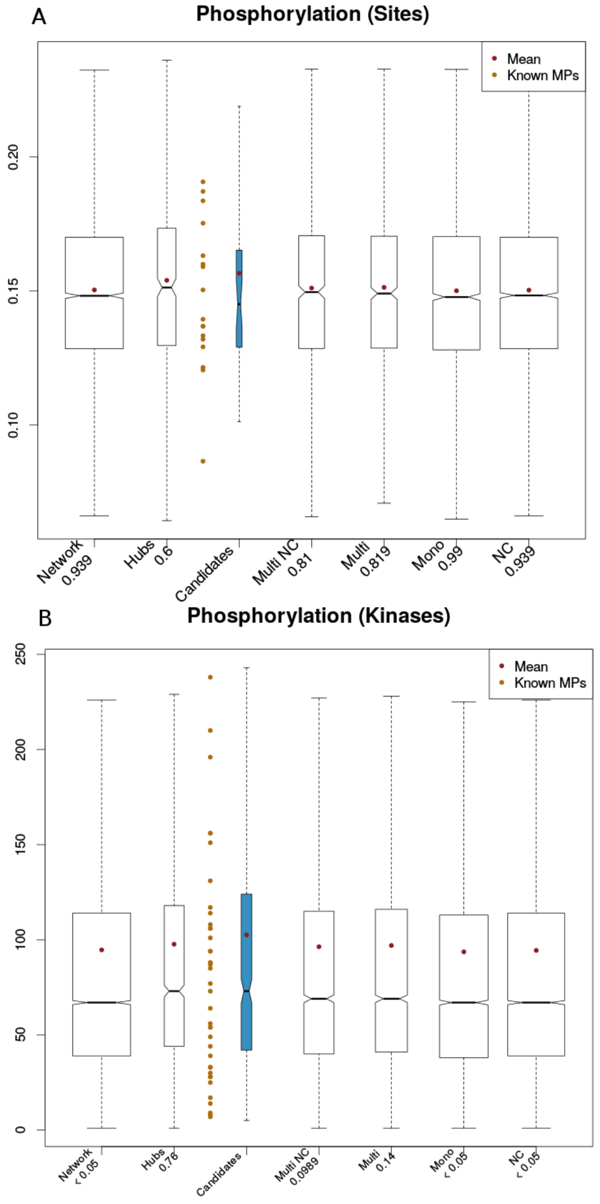

Supplementary Figure 30: Predicted number of different kinases (A) that phosphorylate a protein and phosphorylation sites (B) on a protein divided by the protein's length. Predictions were made using GPS 2.1.

2.12 Supplementary Figure 31: Shortest Paths

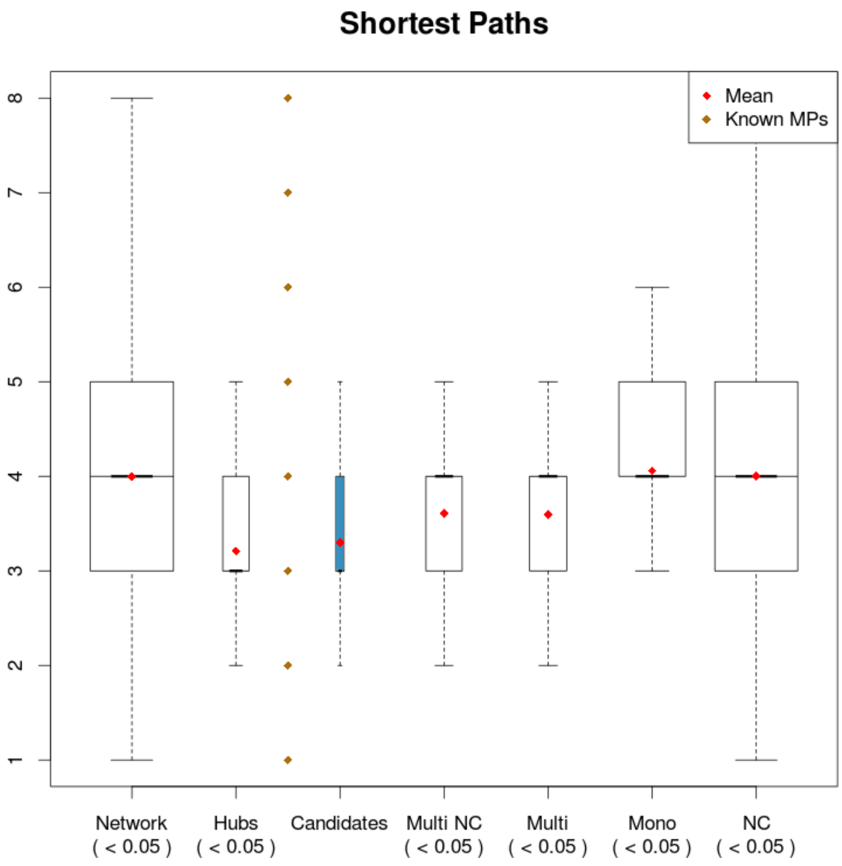

Supplementary Figure 31: Shortest Paths.

3 Supplementary Note

On the choice of similarity measure

In the work presented here, we have chosen not to use semantic similarity measures (SSMs) to assess the similarity of GO terms. Instead, we have developed two new metrics based on GO term co-occurrence as explained in the Methods section. Given the popularity and wide spread use of SSMs, an explanation of this choice is warranted.

We chose not to use semantic similarity measures first because they are particularly ill-suited for the detection of moonlighting or extreme multifunctional proteins. Semantic similarity measures rely on the structure of the Gene Ontology (GO) Direct Acyclic Graph (DAG). They consider two terms to be similar either by measuring their distance as the number of edges connecting them, or by evaluating their information content. These methods can therefore identify semantically similar GO terms, cases where the terms are linked in the structure of the DAG. A trivial example would be "cell death" and "cytolysis" since the latter is a child term of the former. However, the terms "response to tumor necrosis factor" (GO:0034612) and "positive regulation of apoptotic process" (GO:0043065) share no parent terms apart from the root of the ontology and are therefore semantically different. Yet, from a biological perspective they are clearly connected since TNF is a well known inducer of apoptosis. The terms can, therefore, be considered functionally similar since they are different descriptions of the same or tightly linked biological processes. Because our metrics are based on term co-ocurrence, they are independent of the structure of the DAG and can therefore identify such non-semantic, yet functional similarity.

Whether SSMs are the right tool for the job depends entirely on the biological question being asked. SSMs are the tool of choice when, for example, reducing a large list of GO term annotations to a more concise one. On the other hand, as evidenced by their name, semantic similarity measures are geared towards identifying similar terms. In the work presented here, we needed a method for identifying dissimilar pairs. SSMs are not suited for this task since the absence of similarity is not the same as the presence of dissimilarity. In other words, while two terms may not be semantically similar, that does not necessarily make them dissimilar.

To illustrate this particular point, which is crucial for the rationale of our approach, we picked 1000 random Biological Process GO terms pairs (since the BP ontology is used in our work) computed their PrOnto probabilities on the one hand and their SSMs values on the other, and plotted their values (see Figure 1, next page).

In the plots below, each point represents a randomly chosen GO term pair, the y-axis shows one minus the probability returned by PrOnto and the y-axis the semantic similarity value. Because we are plotting 1-PrOnto, high values mean high similarity. Two points are immediately obvious: i) there is no significant correlation between the two measures and ii) all the SSM methods consider only a small minority of terms to be similar whereas the vast majority clusters at 0-0.1. Therefore, using SSM approaches to classify dissimilar functions, as opposed to similar functions, which is what they have been developed for, would result in most term pairs being considered dissimilar.

In addition, we wanted a way to asses term similarity that was i) based on real biological data as opposed to the mathematical abstraction of the DAG and ii) species-specific. Since SSMs are based on the structure of the ontology, they do not take into account the different frequencies of GO terms in different species.

Finally, we also wanted a method that took into account the frequency of interaction between proteins annotated to each of a given pair of terms. Since one measure of the similarity of two biological processes is how much cross talk there is between them, the PrOnto interaction probabilities give yet another dimension that the SSMs cannot offer.

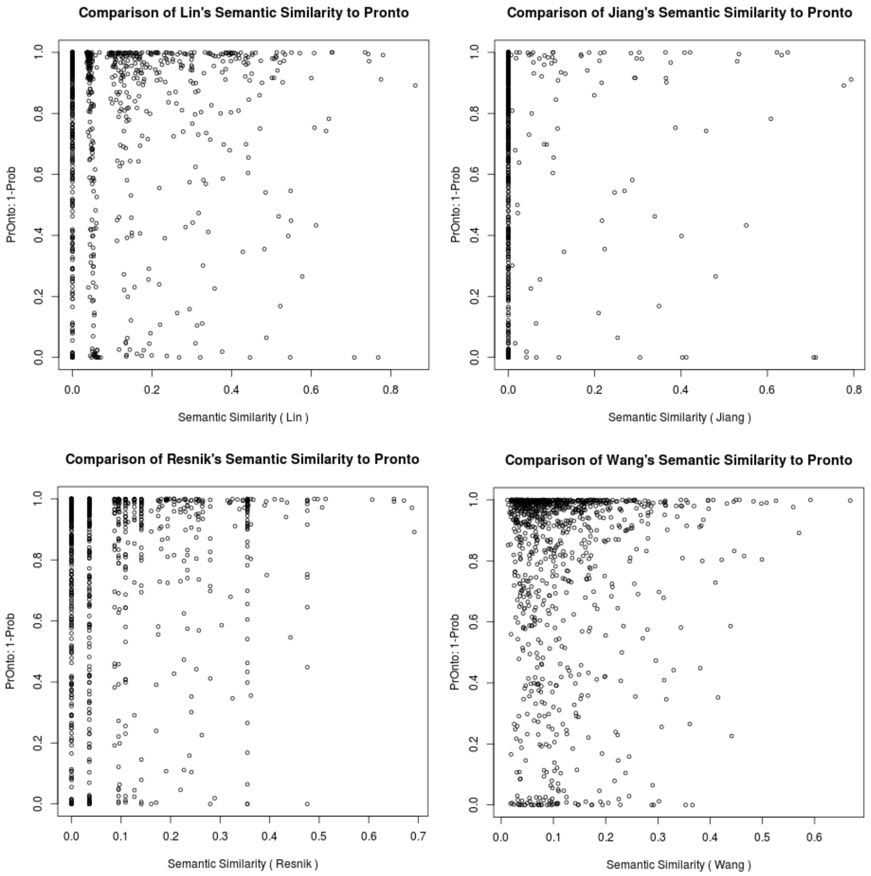

Supplementary Figure 32: Semantic Similarity versus PrOnto probabilities. The y-axis shows 1 minus the PrOnto probability; 1 means “extremely similar” and 0 means “completely dissimilar”. The x-axis shows the semantic similarity scores.
